# Titin kinase ubiquitination aligns autophagy receptors with mechanical signals in the sarcomere

**DOI:** 10.15252/embr.201948018

**Published:** 2021-08-17

**Authors:** Julius Bogomolovas, Jennifer R Fleming, Barbara Franke, Bruno Manso, Bernd Simon, Alexander Gasch, Marija Markovic, Thomas Brunner, Ralph Knöll, Ju Chen, Siegfried Labeit, Martin Scheffner, Christine Peter, Olga Mayans

**Affiliations:** ^1^ Department of Medicine School of Medicine University of California San Diego, La Jolla CA USA; ^2^ Department of Cognitive and Clinical Neuroscience Central Institute of Mental Health Medical Faculty Mannheim Heidelberg University Mannheim Germany; ^3^ Department of Integrative Pathophysiology Medical Faculty Mannheim University of Heidelberg Mannheim Germany; ^4^ Department of Biology University of Konstanz Konstanz Germany; ^5^ Structural and Computational Biology Unit EMBL Heidelberg Germany; ^6^ Integrated Cardio Metabolic Centre (ICMC) Heart and Vascular Theme University Hospital, MedH Karolinska Institutet Huddinge Sweden; ^7^ Bioscience, Cardiovascular Renal & Metabolism BioPharmaceuticals R&D, AstraZeneca Gothenburg Sweden; ^8^ Department of Chemistry University of Konstanz Konstanz Germany

**Keywords:** cellular signalling, mechanotransduction, steered molecular dynamics simulations, ubiquitination, X‐ray crystallography, Cell Adhesion, Polarity & Cytoskeleton, Molecular Biology of Disease, Post-translational Modifications & Proteolysis

## Abstract

Striated muscle undergoes remodelling in response to mechanical and physiological stress, but little is known about the integration of such varied signals in the myofibril. The interaction of the elastic kinase region from sarcomeric titin (A168‐M1) with the autophagy receptors Nbr1/p62 and MuRF E3 ubiquitin ligases is well suited to link mechanosensing with the trophic response of the myofibril. To investigate the mechanisms of signal cross‐talk at this titin node, we elucidated its 3D structure, analysed its response to stretch using steered molecular dynamics simulations and explored its functional relation to MuRF1 and Nbr1/p62 using cellular assays. We found that MuRF1‐mediated ubiquitination of titin kinase promotes its scaffolding of Nbr1/p62 and that the process can be dynamically down‐regulated by the mechanical unfolding of a linker sequence joining titin kinase with the MuRF1 receptor site in titin. We propose that titin ubiquitination is sensitive to the mechanical state of the sarcomere, the regulation of sarcomere targeting by Nbr1/p62 being a functional outcome. We conclude that MuRF1/Titin Kinase/Nbr1/p62 constitutes a distinct assembly that predictably promotes sarcomere breakdown in inactive muscle.

## Graphical abstract

The pseudokinase domain of the titin myofilament is a node for the cross‐talk of mechanical signals and turn‐over pathways in the sarcomere, contributing to a coordinated response to cellular stress.

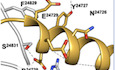

## Introduction

Striated muscle undergoes constant remodelling of its ultrastructure and composition in response to mechanical and physiological stress. Exercise potentiates muscle, while states such as chronic disease, immobilization or nutritional deprivation promote atrophy. Little is known about the molecular mechanisms by which mechanical and physiological signals cross‐talk in the myofibril to result in a combined trophic response. The integration of such varied signals appears to involve the recruitment of cellular proteins onto the contractile sarcomere, which predictably aligns the function of the cell proteome with mechanical cues derived from exercise. The giant protein titin (> 3 MDa) forms intrasarcomeric filaments that span half‐sarcomeres, from Z‐discs to M‐lines, and is known to orchestrate the response of muscle to stress (Kötter *et al*, 2014; Krüger & Kötter, 2016). The interaction of signalling factors, metabolic enzymes and processive proteins with titin in the sarcomere modulates their cellular location and, thereby, their action in the muscle cell in function of mechanical activity (Miller *et al*, 2003; Lange *et al*, 2005; Sheikh *et al*, 2008). The molecular basis of such mechanosensory titin‐based interactions is poorly understood. It is speculated that stretch‐induced conformational changes in titin during muscle function expose cryptic binding sites in the filament that enable association (Lange *et al*, 2005). However, experimental data in support of this mechanism are scarce.

A prominent component of titin to which a mechanosensory function has been attributed is titin kinase (TK), a pseudokinase domain located near the C‐terminus of the protein in the sarcomeric M‐line (Lange *et al*, 2005; Puchner *et al*, 2008; Bogomolovas *et al*, 2014). TK is part of a highly conserved multi‐domain segment of titin with composition: Ig^A168^‐Ig^A169^‐FnIII^A170^‐linker‐TK‐tail‐Ig^M1^ (termed A168‐M1). TK acts as a scaffold that recruits to the sarcomeric M‐line the autophagy receptors Nbr1 and p62 as well as the muscle‐specific E3 ubiquitin ligase MuRF2 (Lange *et al*, 2005). p62 and its interacting partner Nbr1 are adaptor molecules that assemble onto poly‐ubiquitinated protein aggregates, promoting their removal by selective autophagy (Stolz *et al*, 2014). MuRF2 is developmentally regulated and linked to varied trophic events in the myofibril (Perera *et al*, 2012; Peris‐Moreno *et al*, 2020). The assembly of these factors onto TK is thought to be mechanosensitive, being dependent on stretched conformational states of the kinase (Lange *et al*, 2005). In this regard, TK has been attributed elastic properties based on atomic force microscopy (AFM) data and steered molecular dynamics simulations (SMDS) (Gräter *et al*, 2005; Puchner *et al*, 2008; Stahl *et al*, 2011). These studies suggested that a C‐terminal tail extension (CRD) of ∼60 aa length that folds against TK’s active site is able to undergo reversible stretch‐induced unfolding. It was then suggested that the unravelling of the CRD tail in active muscle revealed a cryptic binding site on TK for Nbr1, which interacted with the kinase via its PB1 domain (Lange *et al*, 2005). Nbr1 subsequently recruited p62 and MuRF2 to the kinase (Lange *et al*, 2005). The functional role of the Nbr1/p62 association with TK is unknown, but a sequestration role was speculated for the binding of MuRF2. Released MuRF2 in inactive muscle cells caused the repression of the nuclear serum response factor, thereby inhibiting the transcription of anabolic genes as a consequence of mechanical inactivity (Lange *et al*, 2005).

Notably, the domain tandem Ig^A168^‐Ig^A169^‐FnIII^A170^ (A168‐A170), located N‐terminal to TK, is a receptor site for the E3 ubiquitin ligase MuRF1 (Centner *et al*, 2001; Mrosek *et al*, 2007). MuRF1 transcription is strongly upregulated by atrophic stimuli such as immobilization, denervation, nutritional deprivation and chronic disease, being a critical promoter of muscle loss (Peris‐Moreno *et al*, 2020). The specific role of MuRF1 at the M‐line node is unknown and its functional link to the vicinal TK‐based signalosome has remained unexplored.

In order to investigate signalling cross‐talk mechanisms in the TK locus, we have studied the structure of the multi‐domain TK segment of human titin, analysed its response to stretch using SMDS and explored its functional relation to MuRF1, Nbr1 and p62. The findings indicate that TK is an ubiquitination substrate of MuRF1 and that ubiquitination leads to the assembly of Nbr1 and p62 onto TK. The elastic stretch of TK caused by cytoskeletal forces in the active muscle predictably down‐regulates MuRF1‐mediated titin ubiquitination and, thereby, Nbr1/p62 scaffolding. We propose that in inactive muscle, the MuRF1/titin TK/Nbr1/p62 assembly constitutes a mechanistically distinct signalosome formation on the sarcomeric M‐line, with a likely role in sarcomere breakdown.

## Results and Discussion

### Crystal structure of the titin kinase region

**Figure 1:**
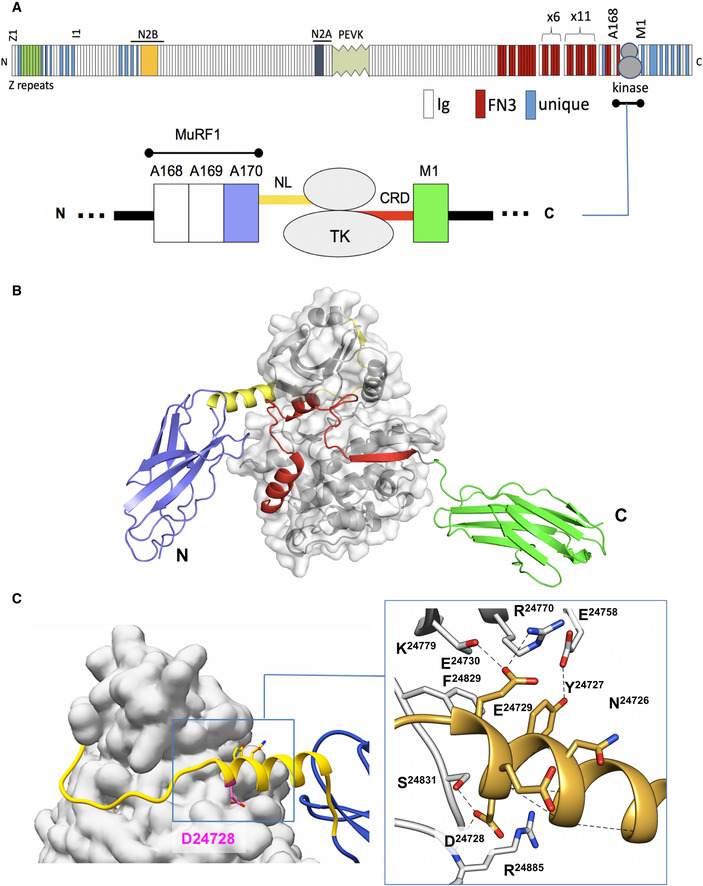
Crystal structure of A170‐TK‐M1 Domain composition of full‐length titin (*top*) and detail of the A168‐M1 kinase region in the sarcomeric M‐line (*bottom*). Domain colour‐coding in the latter is maintained throughout this manuscript. Various components of full‐length titin are annotated to serve as markers. The MuRF1‐binding site in the multi‐domain kinase region is indicated.Crystal structure of A170‐M1.Close‐up view of the packing of the NL sequence against TK (left). Residues in the NYD motif are displayed. The conserved aspartate residue affected by the D24728V exchange in SNP rs200675195 is marked in magenta. Directed interactions established by NYD residues with other parts of the A170‐M1 protein are shown in the inset (right) as dashed lines. These interactions are the primary anchor of the NL element onto TK.

**Figure EV1:**
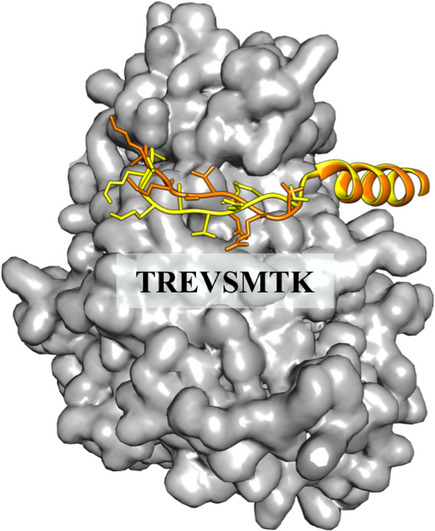
Structural differences in the NL segment of molecular copies of A170‐M1 Copies A and B of molecule A170‐M1 in the crystallographic asymmetric unit closely resemble each other (RMSD_NCS_ = 0.46 Å for 516 Cα atoms). Differences are not observed for any part of the molecular backbone other than an 8‐residue portion of the NL chain that straddles TK. Here, chain A (yellow) and chain B (orange) pack differently onto the kinase domain (grey); the sequence of this segment is shown. The differences are due to lattice packing effects, with each of the NL regions making different contacts with symmetry‐related M1 domains. This observation supports the conclusion that the packing of the NL against TK in this region is weak and can be easily altered, with the NYD motif being the main NL anchor onto TK.

The atomic structure of the Fn^A170^‐NL‐TK‐CRD‐Ig^M1^ multi‐domain kinase region from human titin (termed A170‐M1; Fig 1A) has been elucidated using X‐ray crystallography to 2.4 Å resolution (Table 1). The structure shows the kinase domain, TK, in the context of its flanking elements within the titin chain. A170‐M1 shows a compact arrangement, where TK is enwrapped by its N‐ and C‐terminal extensions (NL and CRD) with domains A170 and M1 projecting from the assembly (Fig 1B). The CRD extension wraps around the larger, C‐terminal kinase lobe, deeply penetrating the catalytic cleft, in agreement with previous structures of the TK domain (Mayans *et al*, 1998; Bogomolovas *et al*, 2014). The newly observed NL sequence is 23‐residue long and displays two structural elements: (i) a prominent frontal α‐helix, 14‐residue long, that forms the junction to domain A170; and (ii) an extended chain that straddles the back of the N‐terminal kinase lobe (Fig 1C). Both helical and extended components pack against the interlobular kinase hinge region. The primary contributor to the packing of the NL tail against TK is a NYD motif (residues 24,726–24,728) within the frontal α‐helix. This motif holds numerous contacts with TK and the CRD (Fig 1C; inset). Of notice are the following: a hydrophobic cluster centred on the NL residue Y24727 (involving residue F24829 in TK, and V25040 and V25041 in the CRD) and the hydrogen bonds held by D24728, an unusually buried aspartate in the interface with the kinase domain (there it binds S24831 and the amino backbone group of R24885). Jointly, these interactions anchor the NL tail onto TK. Notably, the extended NL element (ii) binds TK only loosely. As a consequence, structural differences in this segment are observed between the two molecular copies of A170‐M1 in the asymmetric unit of this crystal form (Fig EV1). This is the only observable difference between non‐crystallographic symmetry copies of the molecule (RMSD_NCS_ = 0.46 Å for 516 Cα atoms).

**Table 1:** Data collection and refinement statistics.

	A170‐M1
**Data collection**	
Space group	*P*2_1_
Cell dimensions	
*a*, *b*, *c* (Å)	63.64, 184.73, 66.11
α, β, γ (°)	90, 116.597, 90
X‐ray beamline	I04‐1 (DIAMOND)
Detector	PILATUS 2M
Wavelength (Å)	0.9173
Resolution (Å)	29.8–2.4 (2.5–2.4)^a^
No. reflections	52256 (6041)^a^
*R*_sym_(I) (%)	11.3 (71.7)^a^
*I*/σ*I*	9.74 (1.75)^a^
CC½	99.1 (69.3)^a^
Completeness (%)	97.9 (98.6)^a^
Redundancy	3.45 (3.45)^a^
**Refinement**	
No. working/free reflections	52,229/2,660
*R*_work_/*R*_free_ (%)	17.32/23.62
No. Atoms	
Protein	8,852
Water	205
R.m.s. deviations	
Bond lengths (Å)	0.009
Bond angles (°)	0.963
Ramachandran plot (%)	
Favoured/allowed	95.89/3.38
Outliers	0.73

^a^ Values in parentheses are for the highest resolution shell.

Interestingly, the compact arrangement of human titin A170‐M1 is reminiscent of that of the kinase region from *C. elegans* twitchin (TwcKR) (von Castelmur *et al*, 2012), the only other member of this kinase family for which a multi‐domain structure is available (a side‐by‐side comparison of A170‐M1 and TwcKR is shown in Appendix Fig S1A and B). This similarity is unexpected as the NL sequences of titin and twitchin do not share length, residue composition or even structure. Strikingly, the NYD motif is conserved in the N‐terminal tails of both kinases, being the only conserved feature (Appendix Fig S1C and D). This points to an important role of this motif in titin‐like kinases.

### MuRF1 ubiquitinates the titin kinase region

**Figure 2:**
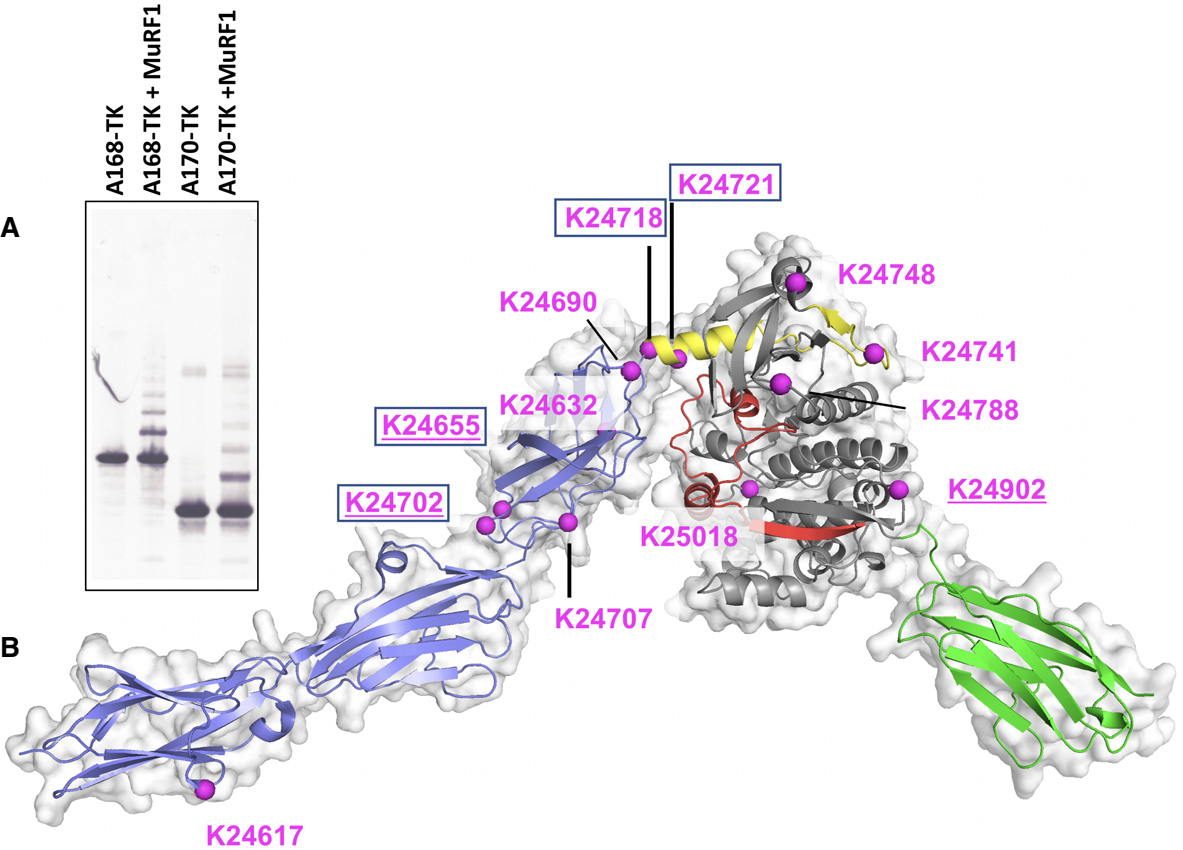
MuRF1‐mediated ubiquitination of the kinase region of titin Anti‐TK Western blot of ubiquitinated titin samples.Reconstruction of the 3D structure of the full‐length kinase region of titin, A168‐M1, achieved by overlapping the structure of A170‐M1 in this work and that of the A168‐A170 previous reported (PDB 2NZI). The presence of a flexible hinge region at the A170‐NL junction can be predicted since the crystal structure of A170‐M1 shows only few and weak interactions between A170 and TK. Otherwise, the A168‐M1 titin segment is highly structured. Lysine residues ubiquitinated by MuRF1 (as identified by mass spectrometry) are marked in magenta. Sites found in denervated limb muscle from mice are boxed (Lang *et al*, 2017), and sites found in other *in vivo* studies are underlined (Wagner *et al*, 2012).

The E3 ubiquitin ligase MuRF1 binds to the domain tandem A168‐A170, N‐terminal to TK (Centner *et al*, 2001; Mrosek *et al*, 2007). The crystal structure of A168‐A170 has been previously reported (Mrosek *et al*, 2007). It overlaps with the structure of A170‐M1 in this study in sharing domain A170, so that the superimposition of A170 in both structures allows for the calculation of a model of the full‐length TK region: A168‐M1. The reconstruction shows that the MuRF1‐binding site, A168–A170, and TK are in immediate spatial proximity as a consequence of the compact folding of the NL sequence (Fig 2). However, no significant interactions exist between A168–A170 and TK, suggesting that these loci are flexibly tethered together. TK is a pseudokinase with so far undetected phosphotransfer catalysis (Bogomolovas *et al*, 2014); thus, MuRF1 is an unlikely substrate of TK. Instead, we asked whether TK might be an ubiquitination target of MuRF1. For this, we performed ubiquitination assays (Fig 2A) using MuRF1 and titin samples (segments A168‐TK and A170‐TK) and identified the modified lysine residues using mass spectrometry. Thirteen ubiquitinated lysines were identified in total (annotated in Fig 2B), which largely localized to the A170‐TK junction. Seven of the sites were vicinal to regulatory elements in titin: namely, three sites (K24655, K24702 and K24707) were located at the junction of domains A169‐A170 proximal to a KTLE loop motif in A169 known to be required for MuRF1 binding (Mrosek *et al*, 2007); three sites (K24690, K24718 and K24721) were at the A170‐TK junction near the NYD motif; one site (K25018) was N‐terminal to the CRD regulatory extension of TK. Notably, five of these ubiquitination sites have been observed in titin samples extracted from mouse muscle (Wagner *et al*, 2012; Lang *et al*, 2017), with four of the sites present in samples from denervated limb muscle that served as an atrophy model with elevated MuRF1 levels (Lang *et al*, 2017; indicated in Fig 2B). Our findings now allow classifying the latter sites as targets of MuRF1.

### Nbr1/p62 association with M‐line titin is ubiquitination‐dependent

**Figure 3:**
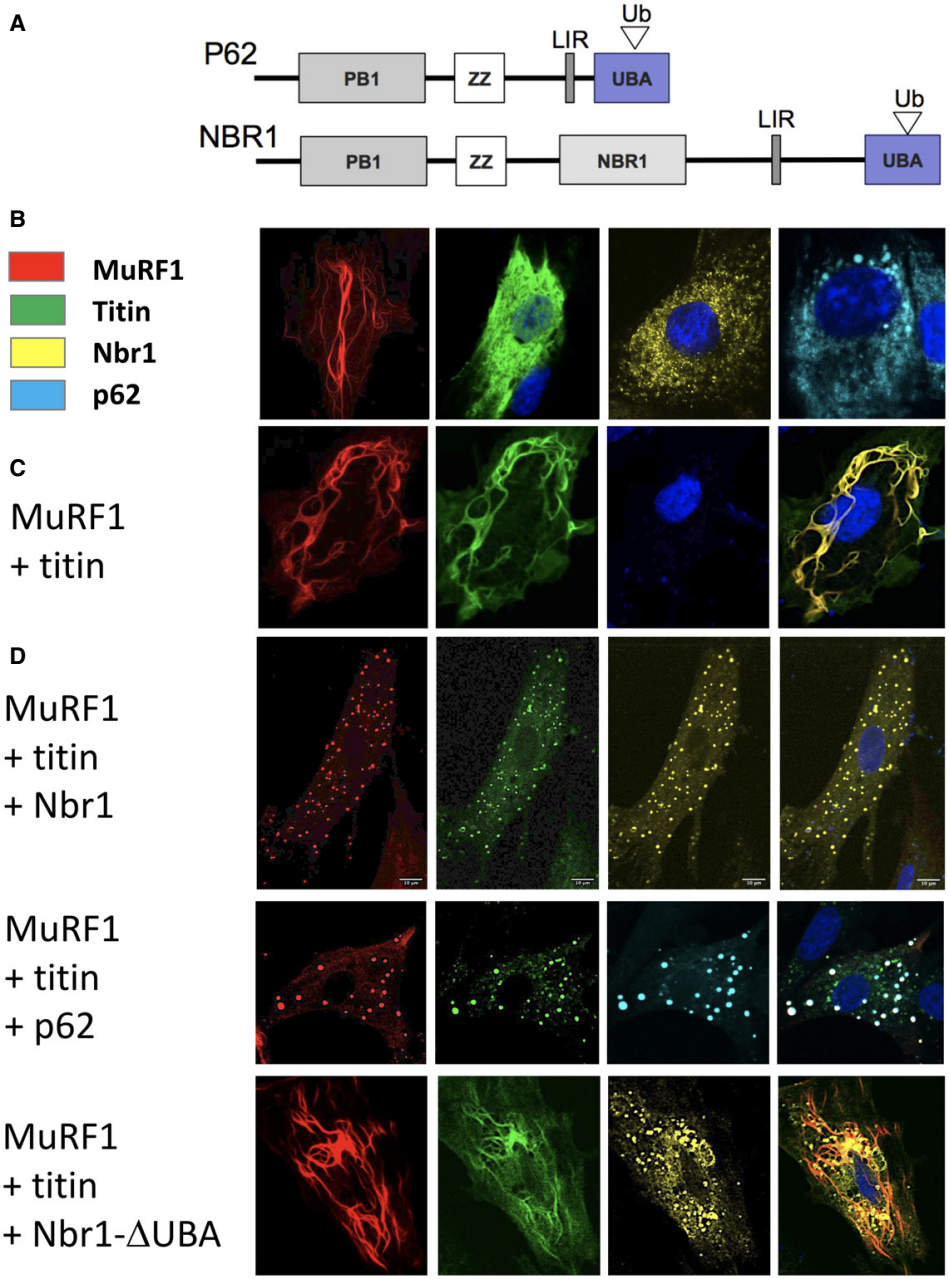
The cellular colocalization of titin A168‐TK with Nbr1 and p62 is MuRF1‐dependent ADomain composition of Nbr1 and p62 proteins, where PB1: Phox and Bem1; ZZ: ZZ finger; NBR1: Next to BRAC1; LIR: light‐chain‐3‐interacting region; UBA: ubiquitin‐binding domain. The ubiquitin‐binding function of the UBA domain is indicated by an open triangle labelled “Ub”.B–DMicrographs of H9C2 cells corresponding to (B) expressions of single species: MuRF1‐mCherry (red), titin EGFP‐A168‐TK (green), Venus‐Nbr1 (yellow) or Cerulean‐p62 (cyan); (C) co‐expression of titin EGFP‐A168‐TK with mCherry‐MuRF1. Neither Nbr1 nor p62 were co‐transfected. The titin fragment and MuRF1 co‐localize forming filamentous formations, which is in agreement with their known complexation; (D) co‐expression of MuRF1‐mCherry (red), titin EGFP‐A168‐TK (green) and Venus‐Nbr1 (yellow) or Cerulean‐p62 (cyan) or respective truncated variants of the latter lacking the ubiquitin‐binding domain (ΔUBA). (Throughout, cell nuclei are stained with DAPI, blue). (Scale bar is 10 μm in all cases).

**Figure EV2:**
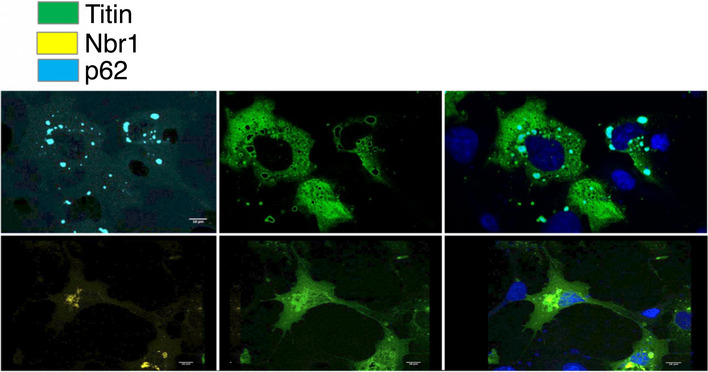
Co‐expression of titin A168‐TK and Nbr1 or p62 in the absence of MuRF1 Titin EGFP‐A168‐TK was coexpressed with Cerulean‐p62 (*upper*) or Venus‐Nbr1 (*lower*) in the absence of MuRF1 in COS7 cells. It can be observed that Nbr1‐ and p62‐rich clusters are depleted of titin A168‐TK, which is spatially excluded. Cell nuclei are stained with DAPI (blue). (Scale bar is 10 μm in all cases).

Nbr1 and p62 have been observed to associate with the sarcomeric M‐line in native myofibrils (Lange *et al*, 2005). As Nbr1 and p62 are known to bind polyubiquitin chains through their ubiquitin‐binding domains (UBA) (Fig 3A; Kirkin *et al*, 2009; Zaffagnini *et al*, 2018), we speculated that MuRF1‐mediated ubiquitination of the TK region would trigger this association (in the absence of stretch‐induced exposure of cryptic binding interfaces). To test this hypothesis, we performed co‐transfection studies of fluorescently labelled A168‐TK, MuRF1, Nbr1 or p62 samples in the cardiac myoblast cell line H9C2 (Fig 3). When expressed in isolation, the titin fragment A168‐TK remained diffused in the cytoplasm (Fig 3B). When coexpressed with MuRF1, A168‐TK and MuRF1 colocalized into typical filamentous formations (Fig 3C), their colocalization being in agreement with the established interaction of these proteins (Centner *et al*, 2001; Mrosek *et al*, 2007). Nbr1 and p62 expressed in isolation exhibited somewhat punctated patterns on a diffuse background as typically observed for these proteins (Paine *et al*, 2005; Kirkin *et al*, 2009; Fig 3B). When titin A168‐TK was coexpressed with Nbr1 or p62 (in the absence of MuRF1), no colocalization of the proteins was observed, with A168‐TK being excluded from the volumes occupied by Nbr1 or p62 (Fig EV2). However, when expressed in the presence of MuRF1, titin A168‐TK colocalized with Nbr1 or p62, respectively, in puncta also containing MuRF1 (Fig 3C). These data suggested a MuRF1‐dependent association of Nbr1 and p62 with the titin TK segment.

**Figure EV3:**
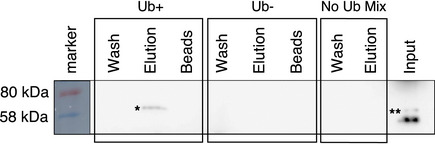
Co‐precipitation experiments using recombinant titin sample Anti‐p62 Western blot of the capture of endogenous p62 from HEK293T cell lysates by recombinant titin GST‐A168‐TK used as bait in pull‐down experiments. Titin samples were incubated with E1/E2/MuRF1 ubiquitination mixtures containing (Ub+) or lacking (Ub−) ubiquitin (as described). Titin samples that had not been exposed to ubiquitination mixtures were also tested (No Ub Mix). Input refers to the whole‐cell lysate containing endogenous p62 (**). Co‐precipitation elution revealed p62 only in the ubiquitinated titin protein fraction (*). These data support the view that p62 complexation with the titin TK segment is mediated by the ubiquitin moiety introduced in titin by MuRF1 and that ubiquitination of the region is required for the interaction to occur.

To test whether the association of Nbr1/p62 with A168‐TK was mediated by the ubiquitin moieties introduced in the titin segment by MuRF1, we performed co‐precipitation experiments using recombinant titin samples, which had been MuRF1‐ubiquitinated *in vitro* and repurified, as bait to capture endogenous p62 and Nbr1 from cell extracts. In this way, endogenous p62 was confirmed to bind selectively to the ubiquitinated titin and not to the unmodified sample (Fig EV3). However, this experimentation was troubled by the intrinsic insolubility of the proteins involved (Paine *et al*, 2005; Kirkin *et al*, 2009), which particularly compromised the monitoring of Nbr1 in this study. Thus, we constructed instead truncated Nbr1 and p62 samples lacking the respective UBA domains (Nbr1‐ΔUBA; p62‐ΔUBA), as these do not associate with ubiquitinated proteins (Kirkin *et al*, 2009; Zaffagnini *et al*, 2018). The truncated variants were then tested in co‐transfection experiments in H9C2 cells. As predicted, UBA domain deficiency abrogated the colocalization of Nbr1 and p62 with titin A168‐TK (titin and MuRF1 continued to mutually associate undisturbed) (Fig 3D). These data supported the view that it is the ubiquitination of titin mediated by MuRF1 that promotes the recruitment of Nbr1 and p62.

Notably, ubiquitin‐mediated scaffolding roles have been proposed for other kinases, for example ubiquitination of LBK1 stabilizes the formation of an active complex with STRAD and MO25 (Lee *et al*, 2015), and polyubiquitination of TAK1 is required for its formation of a complex with TRAF6 and MEKK3 (Yamazaki *et al*, 2009). It is thus conceivable that ubiquitin chains also act as a scaffold in forming the A168‐TK/MuRF1/NBr1/p62 assembly in the sarcomere.

### The NL sequence mediates the mechanical response of the multi‐domain TK region

**Figure 4:**
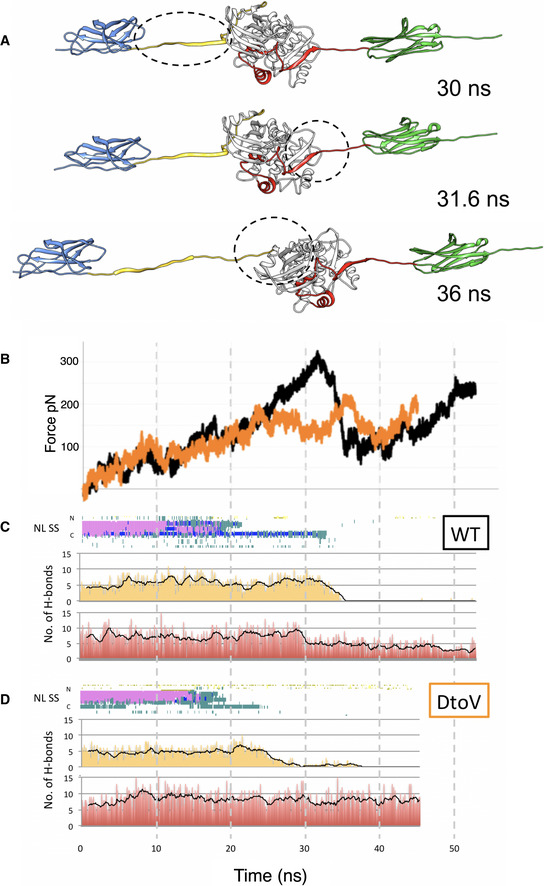
Response to stretch of wild‐type A170‐M1 and its D24728V variant analysed through simulation AStretch‐induced conformational states of A170‐M1 corresponding to main mechanical events. In brief, after 20 ns, the frontal α‐helix in the NL has been lost, at 30 ns the frontal fraction of the NL is completely unfolded, at 31.6 ns the C‐terminal β‐strand in the CRD starts to unravel and by 36 ns, the NL has unwrapped and become completely disassociated from the TK domain. The main force peak corresponds to the unfolding of the “knot” formed by the NYDEE motif in the NL segment, which occurs just before the final complete detachment of the NL. At this point, the kinase N‐lobe started to unfold and the simulations were terminated. Domain colour‐coding as in Fig 1A.BForce versus pulling time curves obtained by SMDS of wild‐type A170‐M1 (black) and A170‐M1^D24728V^ (orange).C, DThe change in secondary structure (SS) composition of the NL segment during the simulation is shown for wild‐type and D24728V proteins. Here, the secondary structure content of NL is shown vertically for each time point. Secondary structure is as defined in VMD (Humphrey *et al*, 1996) where pink, α‐helix; teal, turn; blue, 3_10_ helix; yellow, β‐strand. Histograms showing the number of hydrogen bonds established between regulatory tails (NL, yellow; CRD, red) and the TK kinase domain over the course of the simulation are also shown. Hydrogen bonds were measured using VMD (Humphrey *et al*, 1996) applying distance and angle cut‐offs of 3 Å and 20°, respectively. Values averaged over 1 ns are shown as a black line in the histogram profile. The graphs show that the α‐helical composition of the NL tail is lost early in the simulation of both wild‐type and D24728V models and that, under the simulation conditions, the NL detaches completely from TK but the CRD does not. The differences in the histograms of wild‐type and D24728V models are due to the formation of the resistant “knot” structure in the NL of wild‐type A170‐M1, which causes the CRD to react to stretch by the detachment of its β‐R1 strand. In contrast, the lax NL of the D24728V model prevents stretch from impacting the CRD sequence, which remains largely unaltered throughout the simulation (number of hydrogen bonds remain approximately constant).

To understand how the TK region of titin senses and responds to mechanical stretch and how this might relate to its mediation of protein interactions, we subjected the crystal structure of A170‐M1 to SMDS. In the simulation, the A170‐M1 molecule was fixed at its C‐terminal Cα atom while a force was applied to the N‐terminal Cα atom at a stretching velocity of 5 Å/ns and using a spring force constant of 50 kJ mol^−1^ nm^−2^. The simulation continued until the N‐terminal kinase lobe started to unfold, corresponding to 53 ns simulation time. The resulting force versus pulling time curve and the corresponding molecular transitions are shown (Fig 4A–C; Movie EV1). The results revealed that by 10 ns, the A170‐M1 molecule aligned in the direction of force and A170 extended away from the kinase domain. At this point, unfolding started and proceeded in three primary stages. The first event upon extension (stage 1) was the unfolding of the frontal α‐helix in the NL segment (by 30 ns, the α‐helix had completely unfolded). The easy loss of this element as a first unfolding event was validated in independent simulations that applied a lower spring force constant of 25 kJ mol^−1^ nm^−2^ (over 45 ns simulation). The second stretch event (stage 2) was the unpacking of the C‐terminus of the CRD from TK, specifically the pealing of the CRD β‐strand R1 from TK β‐strand C10 (secondary structure nomenclature as in Mayans *et al*, 1998). Next (stage 3), the conserved NYD motif in the NL sequence detached from TK causing a main force peak and the rest of the NL sequence to unwrap at low force, the NL being thereby fully released from TK. Further stretch caused the N‐terminal kinase lobe to start unfolding. Since unfolded states of TK are unlikely to represent physiological states, simulations were terminated then. At this end point, the NL was fully released as was the CRD β‐R1 strand, but the inhibitory CRD helices αR1 and αR2 remained in place. We speculate that the NL‐unfolded state obtained at low force (where TK, A170 and M1 domains remain structurally intact) represents a physiologically relevant mechanical state of the titin kinase region.

The force curve resulting from SMDS (Fig 4B) features a long shallow slope (virtually a plateau) followed by a single force peak. The shallow slope correlates with the unfolding of the NL α‐helix and the force peak with the detachment of the conserved NYD motif from TK. In helices, the main chain hydrogen bonds align with the helical axis that, in turn, aligns with the force vector during pulling. As a result, helices undergo longitudinal shearing, behaving as constant force springs that display a force plateau and unfold at little force (Rohs *et al*, 1999; Wolny *et al*, 2014). For this reason, helices undergo rapid mechanical folding–refolding transitions and exhibit excellent elasticity. The NYD motif, on the other hand, appears to be a force‐bearing structure. Namely, after the unfolding of the NL α‐helix, a distorted 3_10_‐helical “knot” forms between atoms within the NL ^24726^NYDEE^24730^ motif that then establish multiple contacts to TK (Appendix Fig S2). This knot structure is stable during stretch, withstanding tension and being locked into position until its destruction. Accordingly, the unfolding of the NYDEE knot generates the most prominent force peak in the SMDS extension profile of A170‐M1 (∼32 ns; Fig 4B). Once the knot is released, the rest of the NL unwraps easily from the kinase N‐terminal lobe. We concluded that the NYD motif forms a transient force‐bearing structure that prevents stretch‐induced unfolding from propagating into the kinase domain. Together, the low‐force unfolding of the NL α‐helix and the force‐bearing NYD element combine into a NL architecture well suited to respond to stretch while protecting TK from mechanical damage.

This mechanical model is in excellent agreement with AFM data previously reported for A168‐M2 (Puchner *et al*, 2008; Stahl *et al*, 2011). Those data revealed a first unfolding peak that corresponded to a sequence of ∼9.1 nm length. The sequence was unidentified at that time, but speculated to correspond to features N‐terminal to TK. As described above, the unfolding of linear α‐helices, such as that in TK’s NL, typically occurs at low force and does not yield a force peak, often being undetected by AFM (e.g. Wolny *et al*, 2014; Lanzicher *et al*, 2020). However, our SMDS force profiles reveal that a force peak emerges from the dissolution of the contacts established by the NYD motif. Thus, our simulations are in excellent agreement with AFM observations and explain now their molecular basis. These results are also in best agreement with data from nematode twitchin kinase, the only other member of the family with an NL segment analysed by SMDS (von Castelmur *et al*, 2012). This agreement was unexpected as the NL segments of twitchin and titin diverge noticeably (Appendix Fig S1). The consensus suggests that the NL segment is a primary mechano‐responsive element in this kinase family. Most recently, a report that used FRET to monitor conformational changes in twitchin kinase in freely swimming *C. elegans* has brought support to the view that such stretch‐unfolding is a physiological mechanism that occurs *in vivo* in active muscle (Porto *et al*, 2021).

### NL alterations predictably affect the MuRF1‐mediated ubiquitination pattern of titin

**Figure 5:**
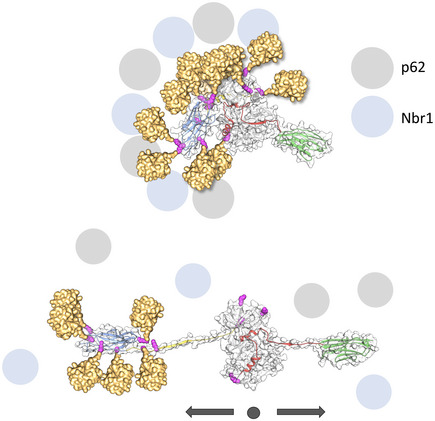
Proposed model of the force‐dependent ubiquitin patterning of titin Hypothetical model of MuRF1‐mediated ubiquitinated titin. Ubiquitin molecules are shown in gold and placed at the ubiquitination sites identified via mass spectrometry in this work. For simplicity, only monoubiquitination is displayed. Stretch‐induced unfolding of the NL sequence would cause the distancing of MuRF1 respect to its target sites in TK, thereby reducing or preventing their ubiquitination. In turn, this might alter the capability of TK to recruit Nbr1 and p62. Domain colour‐coding as in Fig 1A.

The mechanical unfolding of the NL element suggested by SMDS calculations would allow the proximity of the MuRF1‐binding site, A168‐A170, and TK to be regulated dynamically by stretch. Upon stretch‐unfolding, the NL α‐helix would expand from ∼2.1 nm to ∼4.9 nm (when considering the axial distance between adjacent residues in extended conformation as 3.5 Å), thereby more than doubling its length upon a small force input. If the full‐length NL sequence (23‐residue long) became unfolded, the distance between TK and the MuRF1‐binding site would increase to ∼8.0 nm (distance approx. comparable to two serially linked Ig domains). Such stretch‐induced extension could be expected to affect the ability of MuRF1 to ubiquitinate its target sites in the TK segment, leading to a decrease in the ubiquitination levels of this titin locus in an active muscle. It has been recently reported that p62 efficiently targets ubiquitinated proteins that carry at least two polyubiquitin chains consisting of three or more ubiquitin units (Zaffagnini *et al*, 2018). It is, thus, conceivable that a decrease in TK ubiquitination levels might down‐regulate the recruitment of p62/Nbr1 (Fig 5). We speculate that, in net terms, mechanical signals might titrate titin ubiquitination levels and, thereby, modify its scaffolding properties in function of sarcomere activity.

**Figure EV4:**
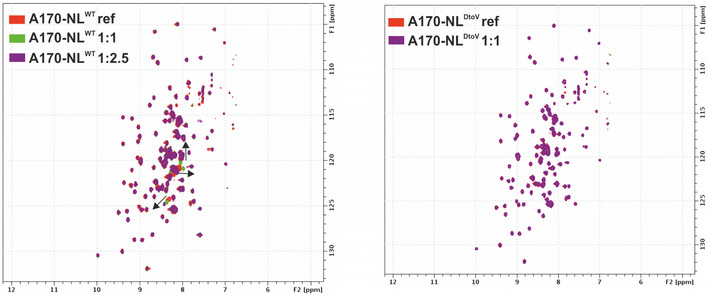
Effect of the D24728V exchange on the A170‐NL/TK interaction HSQC‐monitored titration of A170‐NL (left) with increasing concentrations of TK (red, green, magenta). The major peak shifts are indicated by black arrows and they correspond to residues in and immediately neighbouring the NYD motif (peak assignment in Appendix Fig S4). By comparison, the HSQC spectra of A170‐NL^DtoV^ (right) revealed no peak shifts when overlaying the reference spectrum (red) with that recorded in the presence of TK (1:1 ratio, magenta).

**Figure EV5:**
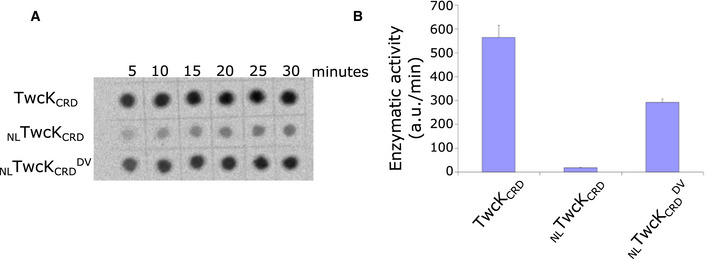
Phosphotransfer activity of TwcK variants A solid‐phase radioassay that used ATP[γ‐^33^P] was employed to quantify the phosphotransfer activity of TwcK variants on a model peptide substrate derived from chicken myosin light chain.Radioactive quantification of the phosphotransfer assay shown in (A). Enzymatic activity is expressed as arbitrary units per minute, where arbitrary unit is defined as the intensity of a blot spot measured with a phosphorimager and quantified using AIDA (Raytest). Histogram bars represent mean values and error bars are +/‐ SD (values derived from three technical replicates). Samples of _NL_TwcK_CRD_ containing both inhibitory regions showed only residual amounts of catalysis. Introduction of a D24728V‐equivalent mutation in this sample (_NL_TwcK_CRD_^DtoV^) increased notably phosphotransfer activity, indicative of a loosening of the NL packing against TwcK.

Currently, no *in vitro* methodologies exist that permit applying stretch to a protein molecule along a defined axis as to induce stretch‐unfolding in conditions that are compatible with downstream biochemical or functional assays. Thus, the generation and testing of the directionally stretched state of A168‐TK could not be performed in this study. Instead, we attempted to engineer mutated A168‐TK variants with a disrupted NL packing to explore whether that would influence the ubiquitination pattern of titin, but variants where poorly viable for experimentation. We sought then a point mutation that could loosen or partly release the NL from TK. For this, we targeted the NYD motif as our structural and SMDS data indicated its importance as NL anchor. Specifically, we chose the exchange D24728V that corresponds to the rare SNP rs200675195 reported in genomic databases enriched in cohorts from patients with heart, lung and blood disorders. The possible association of SNP rs200675195 with muscle disease was supported by our own sequencing of the TK‐encoding titin exon (TTN exon 358) for 200 cardiomyopathy patients as well as our analysis of an available small family tree (Appendix Section S3; Fig S3A and B), bringing support to the relevance of the NYD motif in titin. In D24728V, the small hydrophobic residue valine is incapable of hydrogen bonding through its side chain and can be expected to impair the binding of the NL segment to TK. To validate that the mutated NL chain had indeed a disrupted binding to TK, we (i) performed NMR binding studies between ^15^N‐labelled A170‐NL and unlabelled TK; and (ii) measured the increase in phosphotransfer catalysis in the equally mutated *C. elegans* TwcK homolog that contains an equivalent NYD motif in its NL sequence and for which catalytic protocols are established (von Castelmur *et al*, 2012). In brief, for (i) we performed *in vitro* NMR titration of unlabelled TK with ^15^N/^13^C‐labelled A170‐NL or A170‐NL^DtoV^ samples. We speculated that if the NL sequence and TK interact with each other when in a single polypeptide chain, then upon splitting them into two separate chains, they would still bind to each other. Effectively, chemical shift perturbations in HSQC spectra overlays confirmed that after the split, A170‐NL still interacted with TK (Appendix Fig S4). In good agreement with crystallographic models, the residues that showed chemical shift perturbations were Y24727, D24728, E24729, E24730 and D24732, confirming the central role of this motif in the interaction. However, when TK was titrated with A170‐NL^DtoV^, no chemical shift perturbations were observed, indicating no interaction between both proteins (Fig EV4). In conclusion, NMR data indicated that the D24728V substitution disturbs the NL interaction with TK. This view was also supported by phosphor‐transfer assays (ii). TK appears to be a largely inactive pseudokinase and TK substrates have not been identified to date (Bogomolovas *et al*, 2014). However, the phosphotransfer catalysis of *C. elegans* TwcK on model substrates is well established (Heierhorst *et al*, 1996; von Castelmur *et al*, 2012). The packing of the NL against TwcK is known to inhibit its catalysis, while its truncation leads to TwcK activation (von Castelmur *et al*, 2012). We reasoned that a compromised packing of the NL by the D24728V mutation must also lead to an increase in phosphotransfer catalysis. Effectively, a correspondingly mutated NL‐TwcK‐CRD^DtoV^ sample showed notably increased catalytic activity (Fig EV5 A and B), indicating that the D24728V exchange had disrupted the packing of the NL against the TwcK kinase domain as expected. To complement these results, we also validated the decrease in mechanical response *in silico* of the mutated A170‐M1^D24728V^ by performing SMDS as executed on the wild‐type molecule (Fig 4 B and D; Movies EV2 and EV3). In summary, SMDS, NMR and enzymatic data on D24728V mutated samples confirmed that the residue exchange compromised the NL‐TK interaction and disrupted the mechanosensory role of the NL linker.

We used then A170‐TK^D24728V^ to test whether the conformational alteration of the NL sequence had an effect on its MuRF1‐mediated ubiquitination. A170‐TK^D24728V^ was subjected to ubiquitination assays and the modified lysine residues identified using mass spectrometry as described for the wild‐type. A comparative evaluation of data (from three independent replicates) showed that site K25018 on TK was consistently absent in the mutated sample but present in the wild‐type. The site is located N‐terminally to the CRD sequence, and thus, its modification might have functional consequences for TK. Despite A170‐TK^D24728V^ being a largely inaccurate representative of directionally stretched titin, the result indicates that the ubiquitination of the titin chain is sensitive to conformational changes in the NL segment, bringing support to the view that stretch‐unfolding can lead to a differentially modified titin chain and, in turn, alter the Nbr1/p62 protein interactions it supports (Fig 5).

### Conclusion

The M‐line kinase region of the titin myofilament is an important locus for the cross‐talk of sarcomere stretch signals and cellular turn‐over factors, namely the autophagosomal receptors Nbr1 and p62 and MuRF E3 ubiquitin ligases (Centner *et al*, 2001; Lange *et al*, 2005; Bogomolovas *et al*, 2014). A MuRF2/Nbr1/p62 complex formation onto TK was proposed to occur in active muscle, promoted by the exposure of cryptic binding sites in titin upon sarcomeric stretch (Lange *et al*, 2005). That assembly was initiated by the binding of Nbr1 (through its PB1 domain) to forms of TK lacking the CRD flanking extension. No assembly was observed on full‐length kinase samples and the interaction was independent of the ubiquitination activity of MuRF2. In the current study, we identify the existence of an alternative TK‐based assembly consisting of MuRF1/Nbr1/p62, which occurs in the absence of stretch and that is dependent on MuRF1‐mediated ubiquitination of TK. Ubiquitination promotes then the recruitment of Nbr1/p62 through their UBA domains, which suggests that the interaction is mediated by the ubiquitin chains introduced. MuRF1 is an atrogene that promotes muscle loss. In inactive muscle, the MuRF1 ubiquitination of the TK node could be expected to be maximal as the folded NL would result in a compact form of the titin chain where candidate ubiquitination sites would be proximal to MuRF1. SMDS in this study (supported by early AFM data; Puchner *et al*, 2008) suggest that stretch elicits the unravelling of the NL sequence (Fig 4), separating MuRF1 from its target sites on titin. In stretched states of TK in active sarcomeres, MuRF1 ubiquitination sites could then be displaced and, conceivably, ubiquitination levels decreased. In turn, this would down‐regulate the recruitment of Nbr1/p62 (Fig 5). In net terms, elastic conformational changes in the TK pseudokinase would titrate the ubiquitination output of MuRF1. Thereby, the ubiquitination pattern of M‐line titin in the sarcomere could serve as a readout code of mechanical and physiological stress.

The targeting of M‐line titin by Nbr1/p62, which mediate the autophagy of large protein cargos, in function of MuRF1 could be anticipated to be part of the onset of muscle atrophy. This, as titin molecules deficient in their C‐terminal M‐line are known to result in sarcomere instability and breakdown (Gotthardt *et al*, 2003; Weinert *et al*, 2006; Peng *et al*, 2007; Radke *et al*, 2019). Unravelling the functional consequences of the distinct Nbr1/p62 complexes forming on TK in function of the diverse MuRF1 and MuRF2 ligases (Peris‐Moreno *et al*, 2020) and its consequences for myofibrillar trophicity is a future goal of clinical significance, which will assist the understanding and management of muscle turn‐over.

## Materials and Methods

### Protein production

A codon‐optimized DNA fragment encoding titin domains A170‐M1 (residues 24,622–25,170; reference sequence NP_003310.4) was made synthetically (GeneArt). The DNA was cloned into the pETM‐trx‐1a vector (EMBL) using NcoI/Acc65I restriction sites. This vector adds a His_6_‐tag, a thioredoxin‐tag and a TEV protease cleavage site N‐terminally to the titin insert.

A170‐M1 was expressed in *E.coli* SoluBL21 (Genlantis) grown in Terrific Broth (12 g/l tryptone, 24 g/l yeast extract, 4 ml/l glycerol, 17 mM KH_2_PO_4_, 72 mM K_2_HPO_4_). Cells were grown at 37°C to an OD_600_ of 2–3, then cooled to 18°C and induced with 0.2 mM IPTG overnight. Cells were harvested by centrifugation, washed (50 mM NaH_2_PO_4_ pH 8, 300 mM NaCl) and pelleted. All purification steps were performed on ice or in a refrigerated environment. The pellet was resuspended in T+ buffer (20 mM HEPES pH 8.0, 250 mM NaCl, 5 mM Imidazole, 0.2% Igepal, 2 mM β‐mercaptoethanol, 2 mM PMSF) supplemented with DNAse I and lyzozyme. Cell lysis was by sonification and lysates clarified by centrifugation. The soluble fraction was filtered and applied to Ni^2+^‐NTA resin (Qiagen) pre‐equilibrated with T+ buffer in a gravity flow column. The column was washed with T+, T− (T+ without detergent) buffers and eluted with buffer T− supplemented with 300 mM Imidazole. For TEV cleavage, the buffer was exchanged to 20 mM Tris pH 8.0, 50 mM NaCl, 10% glycerol, 0.5 mM EDTA, 1 mM DTT on a PD10 column (GE Lifesciences) and cleavage carried out overnight at room temperature. Subsequent purification was by anion exchange chromatography followed by gel filtration on a Superdex 75 HL column (GE Lifesciences) in 20 mM HEPES pH 7.8, 50 mM NaCl, 0.25 mM DTT.

Twitchin kinase samples were produced as described (von Castelmur *et al*, 2012). The DtoV exchange in _NL_TwcK_CRD_^DtoV^ was introduced through overlap extension PCR.

All constructs were verified by sequencing.

### Crystal structure determination

Crystals of A170‐M1 were grown in 48‐well crystallization plates (Hampton Research) at 20°C in 2 μl hanging drops obtained by mixing 1:1 the protein stock at a concentration of 14 mg/ml with mother liquor (30% [w/v] PEG 8K, 0.1 M MES pH 6.5). For X‐ray data collection, crystals were cryo‐cooled in LN_2_ in mother liquor supplemented with 30% [v/v] glycerol. Diffraction data were collected on beamline I04‐1 at the Diamond Light Source (Didcot, UK). Data and model refinement statistics are listed in Table 1. Phasing was done by molecular replacement in Phaser (McCoy *et al*, 2007) using TK (PDB entry 1TKI), the A170 FnIII domain (PDB entry 2NZI) and the M1 Ig domain (PDB entry 2BK8) as search models. Model refinement and solvent building was in PHENIX (Adams *et al*, 2011) and manual building in COOT (Emsley *et al*, 2010). Non‐crystallographic symmetry restraints were applied during the early stages of refinement.

### Steered molecular dynamics simulations

Simulations were carried out with GROMACS 5.0.7 (van der Spoel, 2005), using the AMBER99SB‐ILDN force field and periodic boundary conditions. The crystal structure of A170‐M1 (stripped of solvent molecules and alternative rotamers) was solvated with water (TIP3P function) within a box of 9,284 × 10 × 40,029 nm. The total number of atoms was 369,244. Sodium and chloride ions were added to a concentration 0.15 mol/l. The solvated A170‐M1 was energy‐minimized for 1,000 steps using steepest descent. The solvent was then equilibrated for 50,000 steps. The protein and solvent were coupled separately to a 300 K heat bath controlled with a modified Berendsen thermostat (tau = 0.1 ps). The systems were isotropically coupled to a pressure bath at 1 bar maintained using Parrinello‐Rahman coupling (tau = 2 ps, compressibility 4.5 × 10^−5^ bar^−1^). Application of the LINCS (Hess *et al*, 1997) and Settle (Miyamoto & Kollman, 1992) algorithms allowed for an integration time step of 2fs. Short‐range electrostatic and Lennard‐Jones interactions were calculated within a cut‐off of 1.2 nm, the Verlet cut‐off scheme and the neighbour list was updated every 20 steps. The particle mesh Ewald (PME) method was used for the long‐range electrostatic interactions (Darden *et al*, 1999), with a grid spacing of 0.16 nm. A harmonic spring (with a constant of 50 kJ mol^−1^ nm^−2^) was attached to the N‐terminal Cα atom, and the Cα atom of the C terminus was fixed into position. The spring was then moved with a constant velocity of 5 Å/ns along the longest box axis (Z). The system for the DtoV mutant was set up in the same manner except that residue D24728 was replaced for the most common rotamer of valine using COOT (Emsley *et al*, 2010). The average force of the springs was monitored giving the force profiles shown in Fig 4A. Trajectories were analysed with GROMACS (van der Spoel, 2005) and VMD (hydrogen bonds and secondary structure; Humphrey *et al*, 1996).

### In vitro ubiquitination and mass spectrometry

His_6_‐GST‐tagged A168‐TK sample and His_6_‐tagged A170‐TK (2 µg each) were ubiquitinated for 1 h at 37°C in a final volume of 20 µl containing 20 mM Tris–HCl pH 7.5, 5 mM MgCl_2_, 20 mM KCl, 1 mM DTT, 4 mM ATP, 90 nM human recombinant UBE1 (Boston Biochem), 1 µM human recombinant UBcH5c (Boston Biochem), 1.2 µM His_6_‐MBP‐tagged MuRF1, 125 µM human recombinant ubiquitin (Boston Biochem). Reactions were stopped by adding LDS sample buffer, heated to 70°C for 10 min and then run on NuPage 4–12% Bis‐Tris‐Gels (Thermo Fisher). After Coomassie staining, bands corresponding to monoubiquitinated titin fragments were extracted and transferred to ingel digestion (Shevchenko *et al*, 1996). In brief, proteins were reduced (10 mM DTT), alkylated (55 mM IAA) and digested by trypsin (Promega), using an enzyme to protein ratio of 1:100. After overnight incubation, peptides were gradually eluted by increasing amount of acetonitrile prior to sample desalting by stop and go extraction tips (Rappsilber *et al*, 2003).

For mass spectrometry (MS) analysis, peptides were eluted from STAGE tips by solvent B (80% acetonitrile, 0.1% formic acid), dried down in a SpeedVac Concentrator (Thermo Fisher Scientific) and dissolved in solvent A (0.1% formic acid). An UHPLC‐System (EASY‐nLC 1000, Thermo Fisher Scientific) was coupled in line to a Q Exactive HF orbitrap mass spectrometer (Q Exactive HF, Thermo Fisher Scientific) using an electrospray ionization (ESI) source. Peptides were separated based on hydrophobicity by reverse‐phased chromatography using 20 cm C18 silica columns (1.9 µm C18 beads, Dr. Maisch GmbH) and a 150 min gradient with linearly increasing concentration of solvent B over solvent A from 10% to 38% for 125 min and from 38% to 60% for 8 min, followed by washing with 95% of solvent B for 5 min and re‐equilibration with 5% of solvent B. Full MS spectra were acquired in a mass range of 300–1,750 m/z with a resolution of 60,000 at 200 m/z. Tandem mass spectra (MS/MS) were acquired with a resolution of 15,000 at 200 m/z using data‐dependent mode with a loop count of 15 (top15). MS data were processed by MaxQuant 1.5.3.12 (Cox & Mann, 2008) using the Uniprot human database (release 03/2016). The following parameters were used for MaxQuant analysis: maximum of two miss cleavages, mass tolerance of 4.5 ppm for main search and trypsin as digesting enzyme. Carbamidomethylation of cysteines were set as fixed modification. Oxidation of methionine, acetylation of the protein N‐terminus and ubiquitination of lysine were used as variable modifications. For protein identification, only peptides with a minimum of seven amino acids and at least one unique peptide were utilized.

To ensure data consistency and reproducibility, wild‐type and mutant samples were subjected to three comparative replicate experiments that derived from independent ubiquitination assays.

### Cell transfection

The myoblast cell line H9C2 (ATCC^®^ CRL‐1446™) derived from rat myocardium was maintained in Dulbecco modified Eagle’s medium (DMEM) supplemented with 10% foetal bovine serum (FBS), Insulin‐Transferin‐Selenium supplement (ITS‐X) and normocin (InvivoGen). For confocal imaging, cells were seeded on glass coverslips coated with 0.2% gelatine solution in 12‐well dishes and transfected with the following fluorescent protein‐tagged constructs, either alone or in combination: C‐terminally mCherry‐tagged MuRF1; N‐terminally EGFP‐tagged A168‐TK fragment; N‐terminally Venus‐tagged Nbr1; and N‐terminally Cerulean‐tagged p62. Cloning used the In‐Fusion HD Cloning Kit (Clontech). Full‐length MuRF1 was cloned into the pmCherry‐N1 vector (Clontech). A168‐TK was cloned into the pEGFP‐C1 vector (Clontech). For the generation of tagged Nbr1 and p62 constructs, cDNAs were obtained from Origene (Product codes MR225517, MR226105); Venus (Nagai *et al*, 2002) and Cerulean (Rizzo *et al*, 2004) coding DNA fragments were made synthetically. The pcDNA3.1 vector (Invitrogen) was used to express tagged Nbr1 and p62. Cells were transfected using Lipofectamine 2000 (Life Technologies), according to the manufacturer’s instructions. Briefly, 50–200 ng of each plasmid and 0.5 μl Lipofectamine 2,000 per well were diluted in Opti‐MEM (Life Technologies) and added to cells in growth medium. Cells were fixed 24 h post‐transfection with 4% paraformaldehyde in phosphate‐buffered saline (PBS) for 10 min at room temperature, washed 3 × 5 min with PBS and mounted in ProLong Diamond (Life Technologies) mountant. H9C2 cells were imagined on a Leica SP8 confocal system equipped with a 405 nm diode laser and a white light laser tunable to any desired wavelength of excitation between 470 nm and 670 nm, using a 63× oil immersion objective. Optimization of the excitation wavelength and emission range resulted in negligible spectral bleed‐through (< 1%) and spectral deconvolution was not needed. Single channel and composite images were prepared using Fiji (Schindelin *et al*, 2012).

### Co‐precipitation experiments from cell extracts

Pull‐down experiments were performed that used recombinant, *in vitro* MuRF1‐ubiquitinated titin GST‐A168‐TK sample to capture endogenous p62 and Nbr1 from cell extracts. For this, GST‐A168‐TK recombinantly produced in *E. coli* was firstly incubated with E1, UbcH5c and MuRF1 in the presence (Ub+) or absence (Ub‐) of ubiquitin for 5 h at 37°C. GST beads (GE Healthcare) were then incubated with the Ub+ or Ub− mixtures and with human embryonic kidney (HEK) 293T precleared whole‐cell lysates in cell lysis buffer (10 mM Tris–HCl, pH 7.47, 150 mM NaCl, 1% Triton X‐100, supplemented with the Complete™ protease inhibitor cocktail (Sigma‐Aldrich)) for 2.5 h at 4°C with rotation. An additional negative control was performed by incubating GST‐A168‐TK with cell lysates as described above, but without the ubiquitination mixture. Following incubation, beads were washed with cell lysis buffer. Proteins were eluted with 50 mM reduced glutathione pH 8.0. Protein fractions were monitored by SDS–PAGE, and the capture of p62 from lysates was revealed by Western blot. For the latter, SDS–PAGE proteins were transferred to a polyvinylidene difluoride (PVDF) membrane. The membrane was treated with 5% non‐fat milk (Roth) in Tris‐buffered saline Tween 20 (TBST) solution (20 mM Tris–HCl pH 7.5, 1.5 mM NaCl, 0.1% Tween 20), washed three times with TBST and incubated overnight at 4°C with antibodies against p62 (1:1000) (Cell Signalling Technology). The blot was washed three times with TBST and incubated with horseradish peroxidase‐conjugated rabbit anti‐mouse IgG antibody (Cell Signalling Technology) (1:1,500) for 1 h at room temperature, washed and developed with the ECL™ Western Blotting Analysis System (Merck) according to supplier instructions.

### NMR spectroscopy

A170‐NL and A170‐NL^DtoV^ samples were uniformly ^15^N‐ and ^13^C‐labelled in M9 medium with ^15^NH_4_Cl as source of nitrogen and ^13^C‐glucose as carbon source. Protein production was performed as described above, with the exception of gel filtration, which was performed in 20 mM Tris pH 7.0, 50 mM NaCl, 0.5 mM TCEP, 0.2% NaN_3_. For NMR measurements, the sample was concentrated to 1 mM. Titration used unlabelled TK. ^1^H^15^N heteronuclear single quantum correlation (HSQC) spectra were recorded at 22°C in 20 mM Tris pH 7.0, 50 mM NaCl, 0.5 mM TCEP on a Bruker Avance III 700 MHz spectrometer equipped with an HC triple‐resonance probe. Protein backbone assignments were obtained using triple‐resonance experiments HNCA, HNCACB and CBCA(CO)NH. Spectra were processed with NMRPipe (Delaglio *et al*, 1995) and analysed with NMRView (Johnson & Blevins, 1994).

#### *In vitro* phosphorylation assays

The *in vitro* phosphorylation assays in this work were performed as previously described (von Castelmur *et al*, 2012). In brief, phosphorylation was assayed in 20 mM Tris pH 7.4, 10 mM Mg^2+^‐acetate, 0.05% Tergitol‐type NP40, 0.1 mM DTT, 0.2 mg/ml BSA containing 0.4 mM ATP (0.2 µCi/reaction of [γ‐^33^P]ATP), 30 ng/ml TwcK samples and 0.2 mg/ml peptide substrate at 30°C. The peptide substrate was an established model substrate (KKRARAATSNVFS) derived from chicken myosin light chain (Heierhorst *et al*, 1996; von Castelmur *et al*, 2012). At time points, reaction mixture was withdrawn, spotted and phosphoimaged. Images were recorded using a phosphoimager BAS‐5000 (Fujifilm) and quantified using AIDA software (Raytest).

## Author contributions

OM and JB conceptualized the study. JB and BF performed molecular biology, protein production, structural analysis and TwcK catalysis; JRF and CP performed SMDS; BM, JB, MM and AG performed ubiquitination assays and MS analysis; JB and BM performed experimentation in cells and cell lysates; JB and BS carried out NMR analysis; JB and RK performed genomic studies; OM, SL, TB, MS, CP and JC provided reagents, experimental guidance, scientific support and supervision; OM, JB and JRF wrote the manuscript.

## Conflict of interest

The authors declare that they have no conflict of interest.

## Supporting information



Appendix

Expanded View Figures PDF

Movie EV1

Movie EV2

Movie EV3

## Data Availability

Crystal structure coordinates and experimental structure factors have been deposited with the Protein Data Bank (entry 6YGN; https://www.rcsb.org/structure/6ygn). Script files for force‐biased molecular dynamics simulations (including coordinate files, custom index files and.mpd files used) have been deposited with Figshare (https://doi.org/10.6084/m9.figshare.9777620); the files allow for the reproduction of the simulations when using Gromacs 5. Mass spectrometry data and original source files have been deposited with the ProteomeXchange Consortium via the PRIDE partner repository with the dataset identifiers PXD015339 (https://www.ebi.ac.uk/pride/archive/projects/PXD015339) and PXD015340 (https://www.ebi.ac.uk/pride/archive/projects/PXD015340) for titin wild‐type and D24728V samples, respectively.
