# Supplementary material for: Titin kinase ubiquitination aligns autophagy receptors with mechanical signals in the sarcomere
Source: EMBO Rep. 2021 Aug 17;22(10):e48018. doi: 10.15252/embr.201948018 (PMC8490993; doi:10.15252/embr.201948018)
Supplement: Supplementary file 1 — Appendix [file EMBR-22-e48018-s003.pdf]

APPENDIX

Titin kinase ubiquitination aligns autophagy receptors with mechanical signals in the sarcomere

Julius Bogomolovas, Jennifer R. Fleming, Barbara Franke, Bruno Manso, Bernd Simon, Alexander Gasch, Marija Markovic, Thomas Brunner, Ralph Knöll, Ju Chen, Siegfried Labeit, Martin Scheffner, Christine Peter, Olga Mayans

Table of contents:

Appendix Fig S1: Comparison of the multi-domain regions of TK and TwcK

Appendix Fig S2: Force-bearing structure formed by the NYD motif in the NL sequence

Appendix Section S3: Genetic alteration of the conserved NYD motif in the NL segment might be of biomedical significance

Appendix Fig S3: Genomic analysis of variant SNP rs200675195

Appendix Fig S4: A170-NL binds to TK through residues in and vicinal to the NYD motif

Appendix References

Appendix Fig S1: Comparison of the multi-domain regions of TK and TwcK

A Crystal structures of the human titin kinase region in this work (left) and that from *C. elegans* twitchin (PDB: 3UTO) (right) (kinase domains are shown as grey surfaces) (left panel is a redisplay of Figure 1B in the main text).

B Packing of NL tails (yellow) in human TK (left) and *C. elegans* TwcK (right) (left panel is a redisplayed component of Figure 1C in the main text). Residues in the conserved NYD motif are displayed, with the aspartate residue D24728V shown in magenta.

C Sequence composition of TK and TwcK NL extensions. No sequence similarity is evident other than the conservation of the NYD motif. Sequences are coloured according to secondary structure: red, helix; blue, sheet; random coil, black.

D Conservation of the [N/D]YD motif in the family of titin kinases. Shown are sequences from vertebrate titin, mollusc and nematode twitchin, and insect projectin. No other sequence similarity is identifiable in the respective linker sequences.

A

B

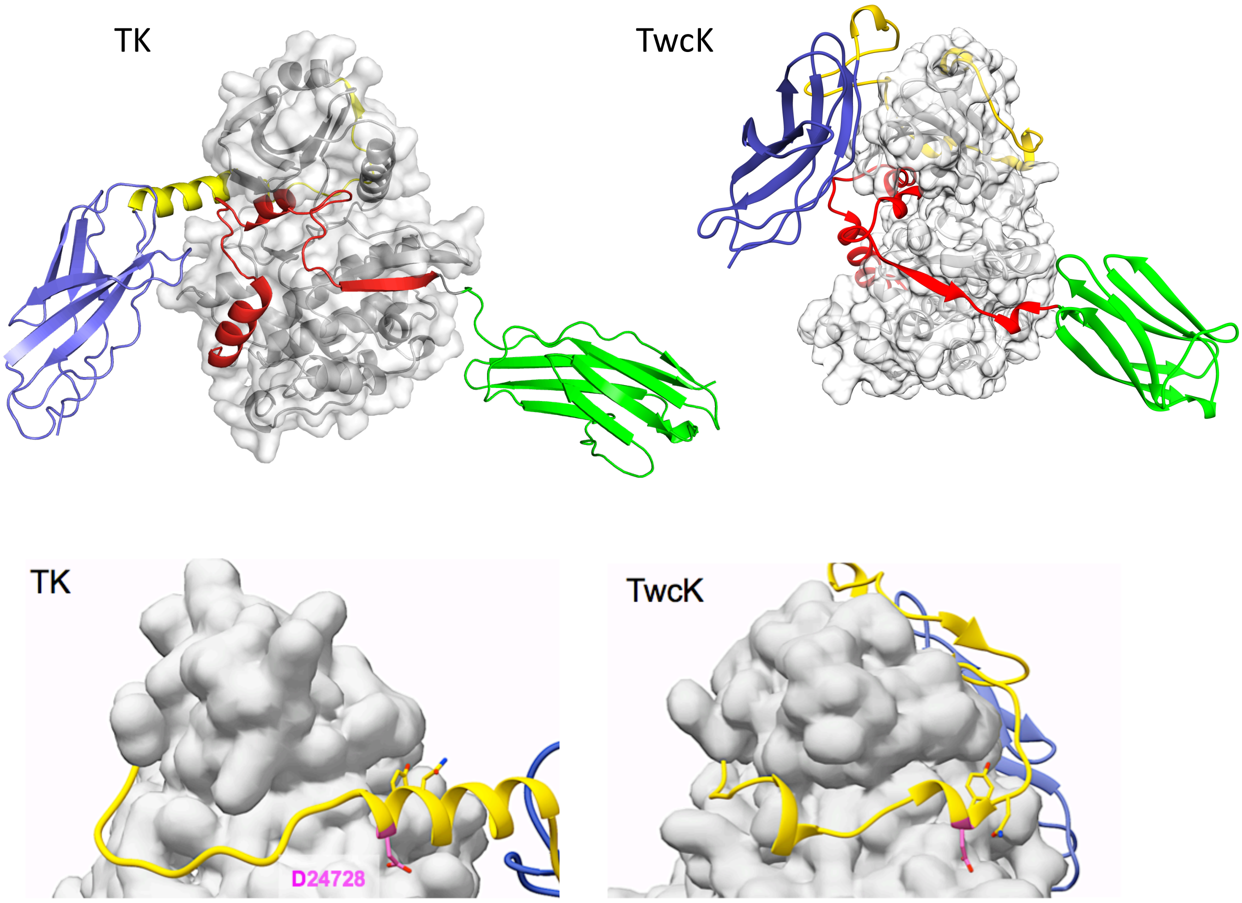

C

```
                                          NYD
TK:                    PTITKEDKTRAMNYDEEVDETREVSMTKA
TwcK:  IPGDERKRRRGYDVDEQGKIVRGKGTVSSNYDNYVFDIWKQYYPQPV
```

D

```
        TITIN__HUMAN        KTRAMNYDEEVDE
        F1RZC8_PIG          KTRAMNYDEEVDE
Titin   F6VG02_HORSE        KTRAMNYDEEVDE
        TITIN__MOUSE        KTRAMNYDDEVDE
        Q7ZZ46_DANRE        KSRVLNYDEEVDD

        UNC22__CAEEL_NEMAT  GTVSSNYDNYVFD
        E1FLZ9_LOALO_NEMAT  GATSDNYDAYVID
Twitchin Q16980_ALPCA_MOLLU GPKINDYDKFYED
        Q86GD6_PROCL_CRUST  KESVDDYDQFVFD
        Q7YT99_MYTGA_MOLLU  GPKVDNYDKYYHD

Projectin B5DRZ6_DROPS_INSEC ADGPKDYDSYVFD
        B0W5W8_CULQU_INSEC  REPIGDYDTYVFD
```

Appendix Figure S2: Force-bearing structure formed by the NYD motif in the NL sequence

A Upon stretch, the NYDEE sequence forms a meta-stable ‘knot’ structure that is created by three primary hydrogen bonds within the NYDEE sequence: *i)* carbonyl oxygen of residue N24726 to main chain nitrogen of E24729; *ii)* N24726 OD1 atom to D24728 backbone nitrogen; and *iii)* Y24727 carbonyl oxygen to E24730 backbone nitrogen. This formation makes extensive contacts to the TK domain and is stretch resistant.

B In the mechanical intermediate state, interactions of relevance with TK are established between residues D24728 and R24885, Y24727 and E25031, E24729 and R24886. Additionally, Y24727 docks into a hydrophobic pocket formed by the TK residues V25041, V25040, F24829 and L24760.

A

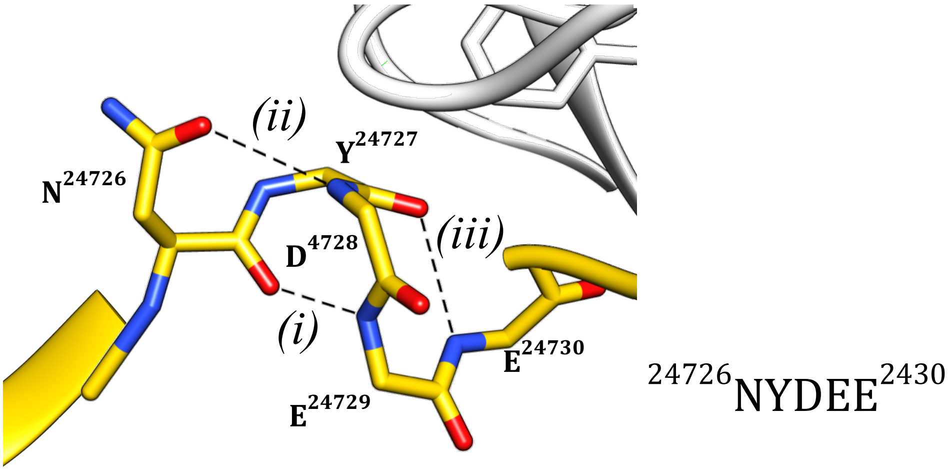

B

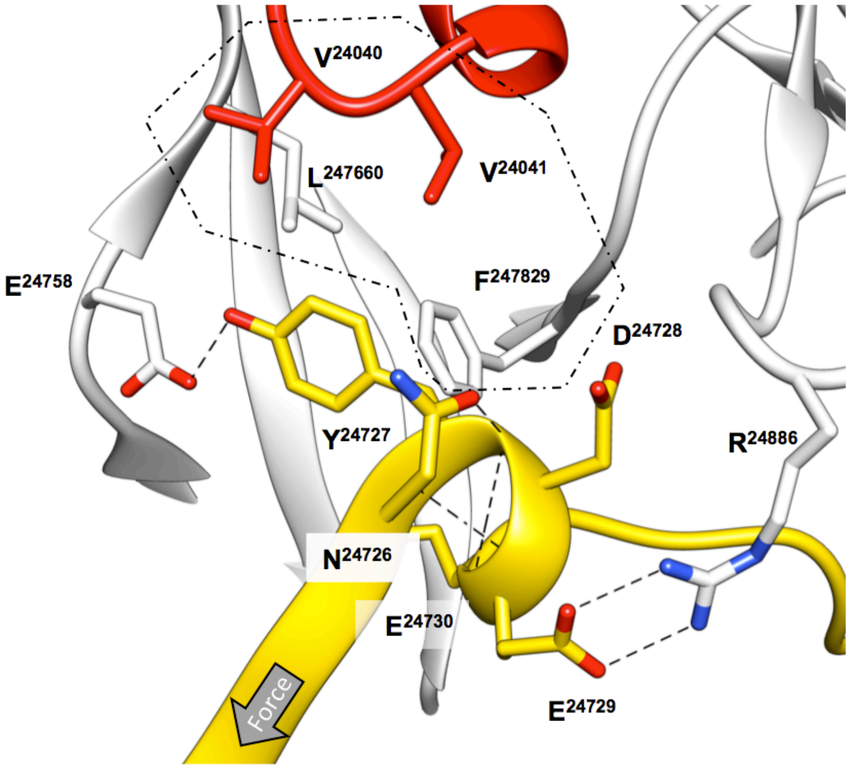

Appendix Section S3: Genetic alteration of the conserved NYD motif in the NL segment might be of biomedical significance

The strong sequence conservation of the NYD motif in the NL segment led us to speculate that its mutation might be detrimental to titin function. To investigate this question, we mined the short genetic variation database dbSNP (https://www.ncbi.nlm.nih.gov/projects/SNP/) and identified SNP rs200675195, which causes the conserved D24728 residue to be exchanged into valine. In a large metacohort (http://gnomad.broadinstitute.org), the exchange has been identified to date in 25 individuals out of 280478 alleles, resulting in a minor allele frequency (MAF) of 0,0089%. In the NHLBI GO Exome sequencing project enriched with patients with heart, lung and blood disorders this allele is 10 times more frequent, being found in 5 heterozygous individuals out of 5956 with MAF of 0.084%. Noteworthy, the exchange D24728V has been interpreted by two submitters in ClinVar (Variation ID:229561) and occurred de novo in two patients with dilated cardiomyopathy (DCM) type 1G and limb-girdle muscular dystrophy type 2J (as recorded in https://www.ncbi.nlm.nih.gov/clinvar/variation/229561).

To further test the possible association of SNP rs200675195 with muscle disease, we performed the sequencing of the TK-encoding titin exon (*TTN* exon 358) in 200 cardiomyopathy patients and sequenced whole exomes of the two patients carrying the SNP rs200675195 to search for other genetic variants associated with DCM. For this, genomic DNA was extracted from the buffy coat using a commercial kit (Qiagen) and exome sequencing was performed commercially (BGI Americas) on two affected subjects. Adapters were ligated to shared genomic DNA, amplified by ligation mediated PCR, purified and hybridized to a custom NimbleGen array for enrichment. Captured fragments were sequenced on a high throughput HiSeq2000 platform. Raw image files were processed by Illumina base calling Software 1.7 for base calling with default parameters and the sequences of each individual were generated as 90bp paired-end reads. Further reads were processed and variants called using two independent workflows: GATK (DePristo et al, 2011) and DNAnexus implemented in DNAnexus cloud-based data analysis and management platform for DNA sequencing data. Variants shared between both subjects were further filtered against dbSNP135 and the 1000 Genomes Project to exclude previously identified disease irrelevant SNPs using wANNOVAR (Chang & Wang,

2012). Genetic variations in identified DCM genes (Meder et al, 2011) were reported. Identical results were obtained using both data processing pipelines. (All experimental protocols were approved by the institutional review board of the host institute, Ethics Commission of the Georg August University of Göttingen, Germany, under the study number: 10/11/04, entitled: "Bedeutung genetischer Faktoren für die Entwicklung einer Herzinsuffizienz").

In both analyzed patient exomes, the set of known DCM associated genes (Norton et al, 2012a; 2012b) had good sequence coverage (Appendix Fig S3A). After variant calling and filtering, two shared heterozygous variants in DCM associated genes were identified: the aforementioned D24728V allele in titin and a second variant, SNP rs201763096 (EtoA), in nexilin, a gene possibly linked to DCM (Hassel et al, 2009). However, the latter SNP has a minor allele frequency of 0.407% according to the gnomAD server (http://gnomad.broadinstitute.org), which is above the threshold for pathogenic DCM mutations (Norton et al, 2012a). Moreover, the nexilin variant was predicted to be benign by PolyPhen2 (Adzhubei et al, 2010) and neutral by PROVEAN (Choi & Chan, 2015). In summary, our sequencing data predict that individuals with the rare SNP rs200675195 in titin have an increased likelihood of suffering from DCM. However, future studies with larger families will be required to formally prove this association and to clarify the relative contributions of SNPs rs200675195 and rs201763096 to DCM in these patients, as compound heterozygotes individuals carrying more than one rare (MAF <1%) sarcomeric allele have a higher risk of adverse cardiovascular events (Bick et al, 2012).

The possible association of SNP rs200675195 with muscle disease was further supported by the analysis of an available small family tree (Appendix Fig S3B).

Appendix Figure S3: Genomic analysis of variant SNP rs200675195

A Average sequencing depth of exons from DCM genes. Data are shown for the two sequenced patients (red and black histograms, respectively).

B Ocurrence of SNP rs200675195 in a small family. The rare SNP rs200675195 causing a D24728V exchange is found in a small family suffering from dilated cardiomyopathy: +/- indicates heterozygosity for the mutation; arrow indicates index patient; grey indicates unknown clinical status; open squares or circles: unaffected; black circles or squares indicate affected; age at death in brackets; slash indicates deceased; roman numbers indicate number of generations studied; arabic numbers indicate patients;

A

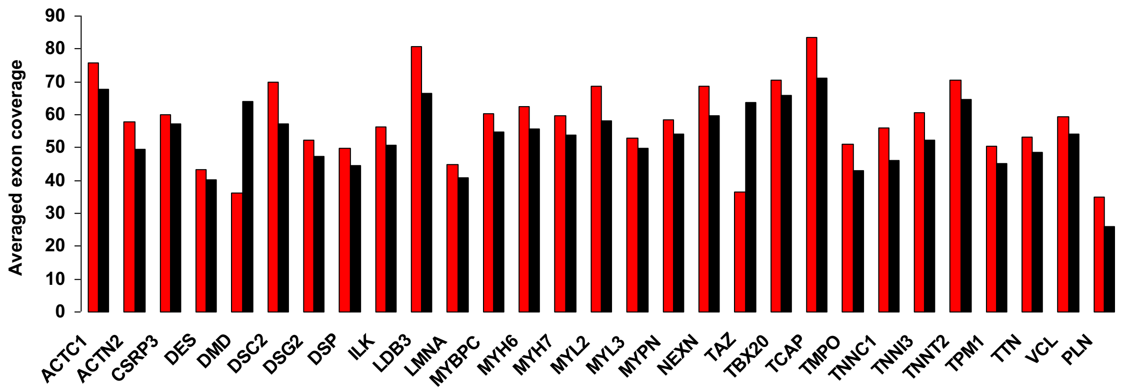

B

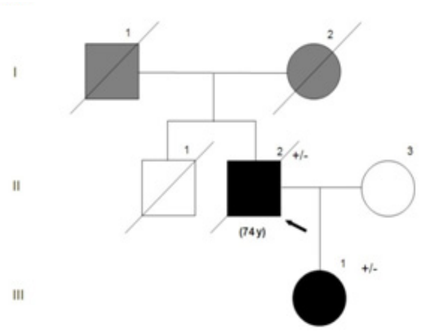

Mutation	Patient	Clinical characteristics	NYHA class
DtoV	II-2	DCM, survived sudden cardiac arrest in 1986	IV
DtoV	III-1	Beginning DCM, sinus tachycardia	I

Appendix Fig S4: A170-NL binds to TK through residues in and vicinal to the NYD motif

Detail of the HSQC-monitored titration of A170-NL (blue) with increasing concentrations of TK (red, magenta, green) showing the major peak shifts.

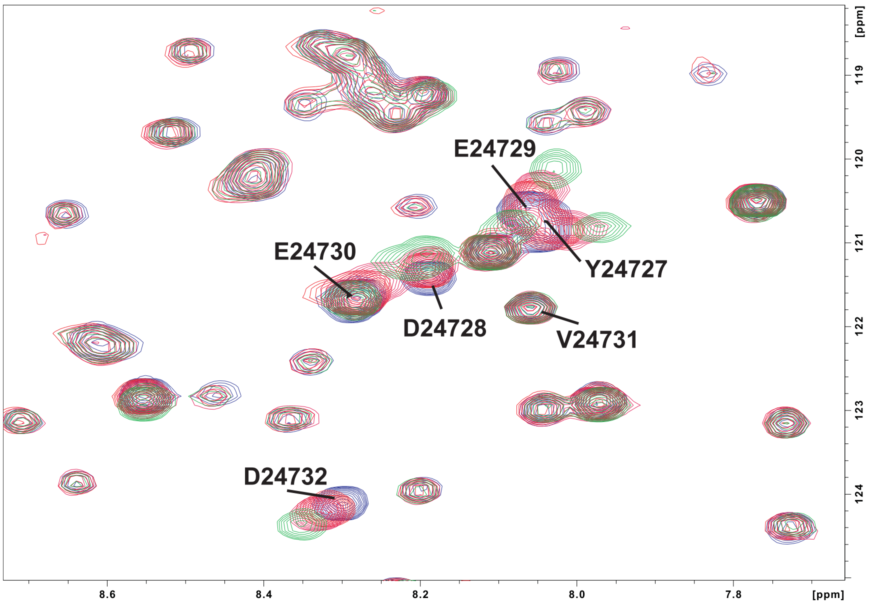

Appendix References

Adzhubei IA, Schmidt S, Peshkin L, Ramensky VE, Gerasimova A, Bork P, Kondrashov AS, Sunyaev SR (2010) A method and server for predicting damaging missense mutations. *Nat Methods* 7, 248-249

Bick AG, Flannick J, Ito K, Cheng S, Vasan RS, Parfenov MG, Herman DS, DePalma SR, Gupta N, Gabriel SB *et al* (2012) Burden of rare sarcomere gene variants in the Framingham and Jackson Heart Study cohorts. *Am J Hum Genet* 91, 513-519

Chang X, Wang K (2012) wANNOVAR: annotating genetic variants for personal genomes via the web. *J Med Genet* 49: 433-436

Choi Y, Chan AP (2015) PROVEAN web server: a tool to predict the functional effect of amino acid substitutions and indels. *Bioinformatics* 31, 2745-2747

DePristo MA, Banks E, Poplin R, Garimella K V, Maguire JR, Hartl C, Philippakis AA, del Angel G, Rivas MA, Hanna M *et al* (2011) A framework for variation discovery and genotyping using next-generation DNA sequencing data. *Nat Genet* 43: 491-498

Hassel D, Dahme T, Erdmann J, Meder B, Huge A, Stoll M, Just S, Hess A, Ehlermann P, Weichenhan D *et al* (2009) Nexilin mutations destabilize cardiac Z-disks and lead to dilated cardiomyopathy. *Nat Med* 15, 1281-1288

Meder B, Haas J, Keller A, Heid C, Just S, Borries A, Boisguerin V, Scharfenberger-Schmeer M, Stähler; P, Beier M *et al* (2011) Targeted Next-Generation Sequencing for the Molecular Genetic Diagnostics of Cardiomyopathies. *Circ Cardiovasc Genet* 4: 110-122

Norton N, Li D, Hershberger RE (2012a) Next-generation sequencing to identify genetic causes of cardiomyopathies. *Curr Opin Cardiol* 27, 214-220

Norton N, Robertson PD, Rieder MJ, Zuchner S, Rampersaud E, Martin E, Li D, Nickerson DA, Hershberger RE (2012b) Evaluating pathogenicity of rare variants from dilated cardiomyopathy in the exome era. *Circ Cardiovasc Genet* 5, 167-174
