# Supplementary material for: Titin kinase ubiquitination aligns autophagy receptors with mechanical signals in the sarcomere
Source: EMBO Rep. 2021 Aug 17;22(10):e48018. doi: 10.15252/embr.201948018 (PMC8490993; doi:10.15252/embr.201948018)
Supplement: Supplementary file 2 — Expanded View Figures PDF [file EMBR-22-e48018-s005.pdf]

Expanded View Figures

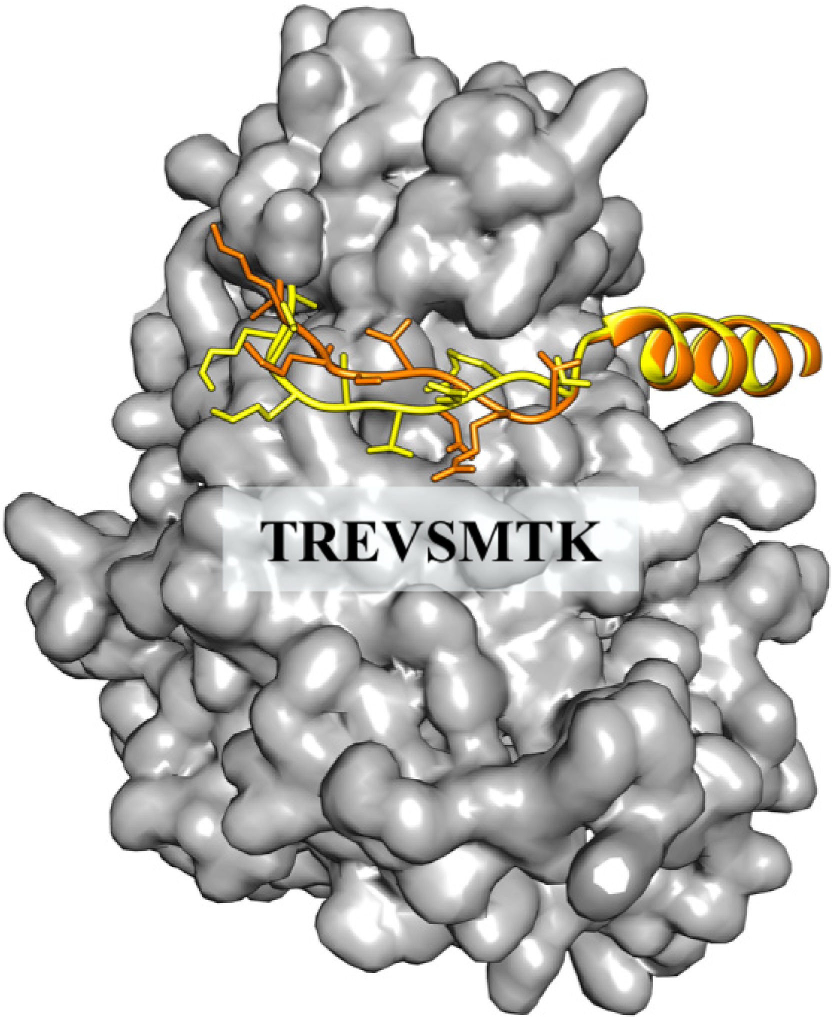

Figure EV1. Structural differences in the NL segment of molecular copies of A170-M1.

Copies A and B of molecule A170-M1 in the crystallographic asymmetric unit closely resemble each other ($RMSD_{NCS}$ = 0.46 Å for 516 Cα atoms). Differences are not observed for any part of the molecular backbone other than an 8-residue portion of the NL chain that straddles TK. Here, chain A (yellow) and chain B (orange) pack differently onto the kinase domain (grey); the sequence of this segment is shown. The differences are due to lattice packing effects, with each of the NL regions making different contacts with symmetry-related M1 domains. This observation supports the conclusion that the packing of the NL against TK in this region is weak and can be easily altered, with the NYD motif being the main NL anchor onto TK.

© 2021 The Authors

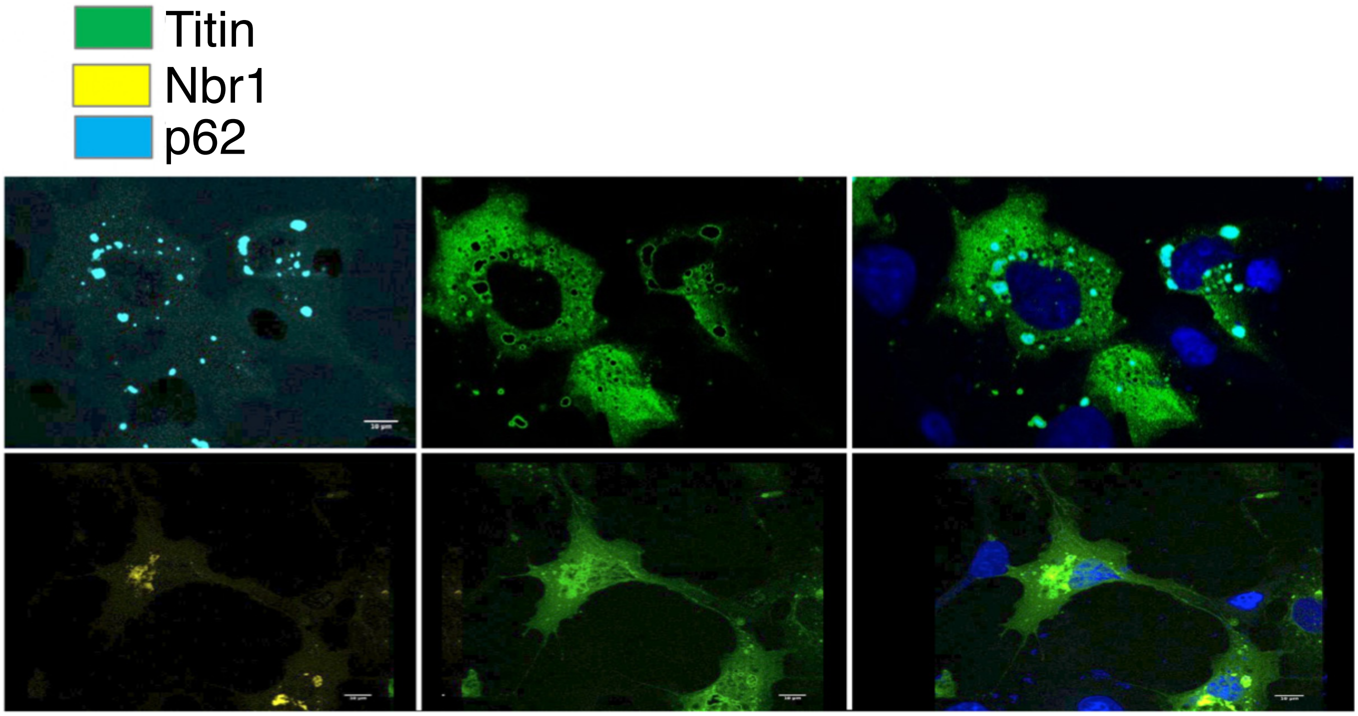

Figure EV2. Co-expression of titin A168-TK and Nbr1 or p62 in the absence of MuRF1.

Titin EGFP-A168-TK was coexpressed with Cerulean-p62 (*upper*) or Venus-Nbr1 (*lower*) in the absence of MuRF1 in COS7 cells. It can be observed that Nbr1- and p62-rich clusters are depleted of titin A168-TK, which is spatially excluded. Cell nuclei are stained with DAPI (blue). (Scale bar is 10 μm in all cases).

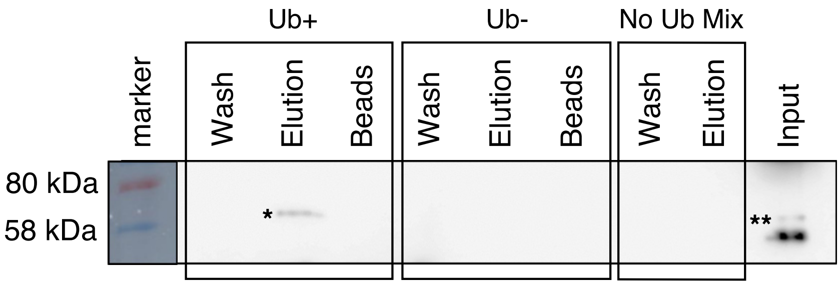

Figure EV3. Co-precipitation experiments using recombinant titin sample.

Anti-p62 Western blot of the capture of endogenous p62 from HEK293T cell lysates by recombinant titin GST-A168-TK used as bait in pull-down experiments. Titin samples were incubated with E1/E2/MuRF1 ubiquitination mixtures containing (Ub+) or lacking (Ub−) ubiquitin (as described). Titin samples that had not been exposed to ubiquitination mixtures were also tested (No Ub Mix). Input refers to the whole-cell lysate containing endogenous p62 (**). Co-precipitation elution revealed p62 only in the ubiquitinated titin protein fraction (*). These data support the view that p62 complexation with the titin TK segment is mediated by the ubiquitin moiety introduced in titin by MuRF1 and that ubiquitination of the region is required for the interaction to occur.

 © 2021 The Authors

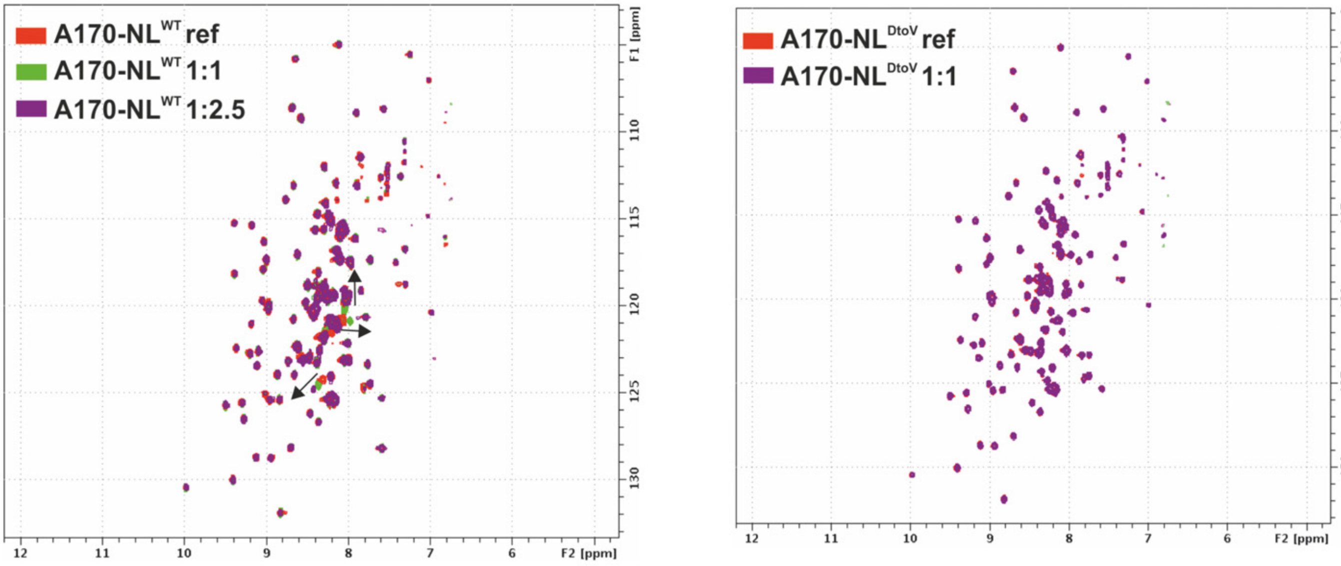

Figure EV4. Effect of the D24728V exchange on the A170-NL/TK interaction.

HSQC-monitored titration of A170-NL (left) with increasing concentrations of TK (red, green, magenta). The major peak shifts are indicated by black arrows and they correspond to residues in and immediately neighbouring the NYD motif (peak assignment in Appendix Fig S4). By comparison, the HSQC spectra of A170-NL^{DtoV} (right) revealed no peak shifts when overlaying the reference spectrum (red) with that recorded in the presence of TK (1:1 ratio, magenta).

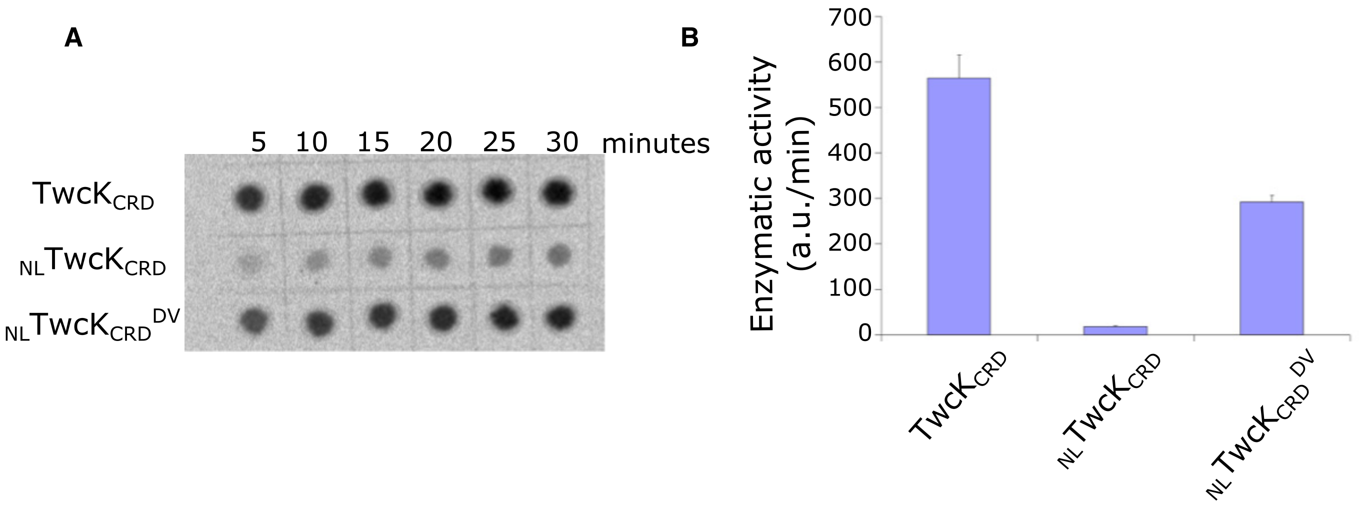

Figure EV5. Phosphotransfer activity of TwcK variants.

A A solid-phase radioassay that used ATP[γ-^{33}P] was employed to quantify the phosphotransfer activity of TwcK variants on a model peptide substrate derived from chicken myosin light chain.

B Radioactive quantification of the phosphotransfer assay shown in (A). Enzymatic activity is expressed as arbitrary units per minute, where arbitrary unit is defined as the intensity of a blot spot measured with a phosphorimager and quantified using AIDA (Raytest). Histogram bars represent mean values and error bars are +/- SD (values derived from three technical replicates). Samples of $_{NL}$TwcK$_{CRD}$ containing both inhibitory regions showed only residual amounts of catalysis. Introduction of a D24728V-equivalent mutation in this sample ($_{NL}$TwcK$_{CRD}$^{DtoV}) increased notably phosphotransfer activity, indicative of a loosening of the NL packing against TwcK.

© 2021 The Authors
