# Supplementary material for: Titin kinase ubiquitination aligns autophagy receptors with mechanical signals in the sarcomere
Source: EMBO Rep. 2021 Aug 17;22(10):e48018. doi: 10.15252/embr.201948018 (PMC8490993; doi:10.15252/embr.201948018)
Supplement: Supplementary file 3 — Movie EV1 [file EMBR-22-e48018-s001.zip › README_Movie_EV1_Caption.docx]

**Movie EV1: SMDS of wild-type A170-M1**

Stretch-induced conformational extension and unfolding of A170-M1. Domain colour coding is as in Fig 1: blue, A170; yellow, NL; white, kinase; red, CRD; and green, M1. In brief, after 20 ns (movie time 00:24) the frontal α-helix in the NL has been lost, at 30 ns the frontal fraction of the NL is completely unfolded, at 31.6 ns (movie time 00:33) the C-terminal β-strand in the CRD starts to unravel, and by 36 ns (movie time 00:35) the NL has unwrapped and become completely disassociated from the TK domain. The lower left hand corner shows the force response profile of A170-M1, i.e. force (kJ mol^-1^ nm^-1^) versus pulling time (ns). The black marker across the force graph indicates the position on the graph of the molecular conformation shown at that time in the movie. The main force peak (movie time 00:34) corresponds to the unfolding of the ‘knot’ formed by the NYDEE motif in the NL segment, which occurs just before the final complete detachment of the NL. At this point, the kinase N-lobe started to unfold and the simulations were terminated.
