# Supplementary material for: Titin kinase ubiquitination aligns autophagy receptors with mechanical signals in the sarcomere
Source: EMBO Rep. 2021 Aug 17;22(10):e48018. doi: 10.15252/embr.201948018 (PMC8490993; doi:10.15252/embr.201948018)
Supplement: Supplementary file 4 — Movie EV2 [file EMBR-22-e48018-s006.zip › README_Movie_EV2_Caption.docx]

**Movie EV2: SMDS of A170-M1^D24728V^**

Stretch-induced conformational extension and unfolding of the mutated A170-M1^D24728V^. Domain colour coding is as before. Unfolding force (kJ mol^-1^ nm^-1^) versus pulling time (ns) is shown in the upper left-hand corner. The black marker across the force graph indicates the position on the graph of the molecular conformation shown at that time in the movie. Here, the NL can be seen to gradually unravel with a shallow force peak, with the complete detachment of the NL achieved by 10 ns (movie time 00:29).
