# Supplementary material for: Titin kinase ubiquitination aligns autophagy receptors with mechanical signals in the sarcomere
Source: EMBO Rep. 2021 Aug 17;22(10):e48018. doi: 10.15252/embr.201948018 (PMC8490993; doi:10.15252/embr.201948018)
Supplement: Supplementary file 5 — Movie EV3 [file EMBR-22-e48018-s004.zip › README_Movie_EV3_Caption.docx]

**Movie EV3: Comparison of the simulated unfolding of wild-type A170-M1 and mutated A170-M1^D24728V^**

Structures were aligned at the first frame. The wild-type form is coloured completely white, A170-M1^D24728V^ is coloured by domain composition as before. NYDEE residues are shown as sticks. Movie time 00:46 shows the key point where NYD ‘knot’ remains for the wild-type A170-M1 but has been lost by the mutant A170-M1^D24728V^.
