# Supplementary material for: Titin kinase ubiquitination aligns autophagy receptors with mechanical signals in the sarcomere
Source: EMBO Rep. 2021 Aug 17;22(10):e48018. doi: 10.15252/embr.201948018 (PMC8490993; doi:10.15252/embr.201948018)
Supplement: Supplementary file 6 [file EMBR-22-e48018-s002.pdf]

EMBO reports

Titin kinase ubiquitination aligns autophagy receptors with mechanical signals in the sarcomere

Julius Bogomolovas, Jennifer Fleming, Barbara Franke, Bruno Manso, Bernd Simon, Alexander Gasch, Marija Markovic, Thomas Brunner, Ralph Knöll, Ju Chen, Siegfried Labeit, Martin Scheffner, Christine Peter, and Olga Mayans
DOI: 10.15252/embr.201948018

Corresponding author(s): Olga Mayans (Olga.Mayans@uni-konstanz.de)

Review Timeline:

Submission Date:	28th Feb 19
Editorial Decision:	26th Apr 19
Revision Received:	18th Apr 21
Editorial Decision:	27th May 21
Revision Received:	7th Jul 21
Accepted:	19th Jul 21

Editor: Deniz Senyilmaz Tiebe

Transaction Report:

(Note: With the exception of the correction of typographical or spelling errors that could be a source of ambiguity, letters and reports are not edited. Depending on transfer agreements, referee reports obtained elsewhere may or may not be included in this compilation. Referee reports are anonymous unless the Referee chooses to sign their reports.)

1st Editorial Decision 26th Apr 2019

Dear Dr. Mayans

Thank you for submitting your manuscript for consideration by EMBO Reports. I would like to apologize for this unusual delay in our decision process. Three referees agreed to review the manuscript, two of which returned their reports. The third referee, on the other hand, has not returned his/her report but given the present set of referee reports I can take a decision at this stage.

Please note that this is a preliminary decision made in the interest of time, and that it is subject to change should the third referee offer very strong and convincing reasons for this. If/When we receive the final report on your manuscript, we will forward it to you as well.

As you can see, both referees express interest in the analysis. However, they also raise concerns that need to be addressed in full before we can consider publication of the manuscript here. In particular, both referees require additional support into the functional relevance of titin ubiquitination and better evidence supporting that ubiquitination of titin recruits Nbr1 and p62, not just stretch induced exposure of the residues. Moreover, both referees would like to see the mass-spec data.

Should you be able to address these criticisms in full, we could consider a revised manuscript. I do realize that addressing all the referees' criticisms will require a lot of additional time and effort and be technically challenging. I would therefore understand if you wish to publish the manuscript rapidly and without any significant changes elsewhere, in which case please let us know so we can withdraw it from our system.

If you decide to thoroughly revise the manuscript for EMBO Reports, please
revise your manuscript with the understanding that the referee must be fully addressed and their suggestions taken on board. Please address all referee concerns in a complete point-by-point response. Acceptance of the manuscript will depend on a positive outcome of a second round of review. It is EMBO Reports policy to allow a single round of revision only and acceptance or rejection of the manuscript will therefore depend on the completeness of your responses included in the next, final version of the manuscript.

We generally allow three months as standard revision time. As a matter of policy, competing manuscripts published during this period will not negatively impact on our assessment of the conceptual advance presented by your study. However, we request that you contact the editor as soon as possible upon publication of any related work, to discuss how to proceed. Should you foresee a problem in meeting this three-month deadline, please let us know in advance and we may be able to grant an extension.
You can submit the revision either as a Scientific Report or as a Research Article. For Scientific Reports, the revised manuscript can contain up to 5 main figures and 5 Expanded View figures. If the revision leads to a manuscript with more than 5 main figures it will be published as a Research Article. If a Scientific Report is submitted, these sections have to be combined. This will help to shorten the manuscript text by eliminating some redundancy that is inevitable when discussing the same experiments twice. In either case, all materials and methods should be included in the main manuscript file.

Supplementary/additional data: The Expanded View format, which will be displayed in the main

HTML of the paper in a collapsible format, has replaced the Supplementary information. You can submit up to 5 images as Expanded View. Please follow the nomenclature Figure EV1, Figure EV2 etc. The figure legend for these should be included in the main manuscript document file in a section called Expanded View Figure Legends after the main Figure Legends section. Additional Supplementary material should be supplied as a single pdf labeled Appendix. The Appendix includes a table of content on the first page with page numbers, all figures and their legends. Please follow the nomenclature Appendix Figure Sx throughout the text and also label the figures according to this nomenclature. For more details please refer to our guide to authors.

When preparing your letter of response to the referees' comments, please bear in mind that this will form part of the Review Process File, and will therefore be available online to the community. For more details on our Transparent Editorial Process, please visit our website: http://emboj.embopress.org/about#Transparent_Process

Regarding data quantification, please ensure to specify the name of the statistical test used to generate error bars and P values, the number (n) of independent experiments underlying each data point (not replicate measures of one sample), and the test used to calculate p-values in each figure legend. Discussion of statistical methodology can be reported in the materials and methods section, but figure legends should contain a basic description of n, P and the test applied. Please also include scale bars in all microscopy images.

We now strongly encourage the publication of original source data with the aim of making primary data more accessible and transparent to the reader. The source data will be published in a separate source data file online along with the accepted manuscript and will be linked to the relevant figure. If you would like to use this opportunity, please submit the source data (for example scans of entire gels or blots, data points of graphs in an excel sheet, additional images, etc.) of your key experiments together with the revised manuscript. Please include size markers for scans of entire gels, label the scans with figure and panel number, and send one PDF file per figure.

When submitting your revised manuscript, we will require:

- a complete author checklist, which you can download from our author guidelines (http://embor.embopress.org/authorguide#revision). Please insert page numbers in the checklist to indicate where the requested information can be found.
- a letter detailing your responses to the referee comments in Word format (.doc)
- a Microsoft Word file (.doc) of the revised manuscript text
- editable TIFF or EPS-formatted figure files in high resolution
(In order to avoid delays later in the publication process please check our figure guidelines before preparing the figures for your manuscript:
http://www.embopress.org/sites/default/files/EMBOPress_Figure_Guidelines_061115.pdf)
- a separate PDF file of any Supplementary information (in its final format)
- all corresponding authors are required to provide an ORCID ID for their name. Please find instructions on how to link your ORCID ID to your account in our manuscript tracking system in our Author guidelines (http://embor.embopress.org/authorguide).

We would also welcome the submission of cover suggestions, or motifs to be used by our Graphics Illustrator in designing a cover.

As part of the EMBO publication's Transparent Editorial Process, EMBO reports publishes online a Review Process File to accompany accepted manuscripts. This File will be published in conjunction

with your paper and will include the referee reports, your point-by-point response and all pertinent correspondence relating to the manuscript.

You are able to opt out of this by letting the editorial office know (emboreports@embo.org). If you do opt out, the Review Process File link will point to the following statement: "No Review Process File is available with this article, as the authors have chosen not to make the review process public in this case."

I look forward to seeing a revised version of your manuscript when it is ready. Please let me know if you have questions or comments regarding the revision.

Yours sincerely,

Deniz Senyilmaz Tiebe

Deniz Senyilmaz Tiebe, PhD
Editor
EMBO Reports

Referee #1:

Revision 2019-04-01; EMBO reports Titin ubiquitination causes the scaffolding of autophagosomal receptors onto the sarcomere
Bogomolovas et al. performed a structural analysis of titin domains, which have been previously associated with Murf1, Nrb1 and p62 binding. This structural analysis is certainly very insightful and allows wide speculations about the functional behavior of these domain upon stretch/mechanical forces. Further, by in-vitro ubiquitylation assay, the authors were able to identify ubiquitylation sites on titin as targets for Murf1. Using SMDS, the authors in turn simulate folding of this titin domain upon stretching and characterize potential opening sites. These opening site is reported to contain a human SNP which has been linked to cardiac diseases. Mutagenesis were used to introduce the SNP variation in order to identify changes in titin domain ubiquitylation. Overall, the authors propose an altered mechanosensory mechanism of higher complexity as previously thought.
While the structural data were quite convincing, other parts of the manuscript unfortunately displayed weaknesses that need to be addressed.
The authors argue convincingly that titin is unlikely to be transported to protein degradation sites and that protein degradation is more likely to occur through the recruitment of ubiquitin binding protein. However, the motivation of the subsequent experiments is not clear as it only partially addresses the underlying scientific question.
Based on the EMBO manuscript submission guidelines, proteomics data should be submitted to a publicly accessible database, typically PRIDE. In addition, all identified sites should be documented in a suppl. table with relevant MS data to judge the quality of the MS data and modified peptides.
The authors claim an absence of ubiquitylation in the D24728V mutant at a specific site but do not provide experimental evidence for this.
A central claim of this manuscript describes titin not only as a sequestering protein, but also as a target of further modification with other functions. While the modification of titin is rather obvious, it is unclear what these new functions may be and the manuscript in its current form do not shed light onto this question. Ubiquitylation of titin may just change sequestration targets by creating binding sites for different types of proteins, which is per se not new. Detailed characterization of the

ubiquitylation site is advisable in particular with respect to protein stability. However, this might be a challenge for the giant protein titin and will be hard to address.
The authors suggest that ubiquitylation recruits Nbr1 and p62 to titin and not stretch-induced exposure of binding sites. A supportive experiment would be useful to show that stretched titin (or equivalent construct) does not bind to Nbr1 or p62. Supporting evidence is lacking and co-localization studies with the unstretched A168-TK construct cannot exclude that Nbr1 and p62 are recruited to titin. It might be helpful to perform an immunoprecipitation experiment with ubiquitylation titin to identify the ubiquitylation-interaction of titin with Nbr1 and p62 via mass spectrometry. The authors propose that Nbr1 and p62 are recruited titin via their ubiquitin binding domain. Importantly, while the A168-TK showed diffuse expression, co-expression with Murf1 leads to A168-TK accumulation in large aggregates (Fig 3c) with remarkable shapes, suggesting for a change in localization of A168-TK itself. One obvious explanation is that A168-TK gets ubiquitylated and subsequently recruited to sites of protein degradation. Conversely, ubiquitin binders Nbr1 and p62 are recruited to these sites as well, independently of A168-TK. Co-localization is not sufficient to show binding of Nbr1 and p62 to A168-TK.
The authors describe that the A170-TKD24728V sample was subjected to ubiquitylation assays and modified lysine residues were identified by MS. The author should show the results from three independent replicates with fold changes, statistical relevant values and MS data.
The authors point out correctly, that endogenous titin is static and rather immobile. However, experiments are done with small constructs covering only one part of titin, which is acceptable given the size of full length titin. However, the authors should address experimentally or carefully discuss, why ubiquitylation of a diffusible protein construct has the same or a similar effect compared to the ubiquitylation of the entire titin.
The authors propose a dynamic regulation of ubiquitylation upon stretch, supported by SMD simulation analysis. However, in-vitro ubiquitination data (Fig2) show that stretch is not required for efficient ubiquitylation. This point should be at least discussed.
Minor points:
For reader convenience, all abbreviations should be clarified. Fig.1A requires improvements. In principle, pieces of information that come up later in the manuscript should be displayed in this panel. This includes: (i) all motifs (ii) the localization of detected ubiquitylation sites (iii) an overview of full length titin, which certainly helps to better orient within this huge protein.
The authors are advised to consistently use the term "mass spectrometry" rather than "mass spectroscopy". In addition, the authors may discuss how many ubiquitylation sites are unknown and which of them have been observed in in-vivo studies using the given reference. An overlap to known sites from the phosphosites.org database could also be useful. The authors shifted several important findings to the supplement, which makes the manuscript difficult to follow. Along these lines, some experiments were obviously performed, but not described or discussed in the manuscript.

Referee #2:

The study by Bogomolovas et al is well designed and executed, providing important new information on the role of titin's extreme COOH-terminus including a pseudokinase domain and the structural elements that flank it in sensing and transmitting mechanical signals via a multiprotein complex consisting of Nbr1/p62 and MURF1.

Major concerns:

1. Ubiquitination experiments: Given that these experiments were done in vitro, the authors need to comment on how confident they are about all the identified sites possibly undergoing ubiquitination in vivo. Also, were the common sites identified in both constructs by mass spec? This needs to be clarified/included.

2.Overexpression studies n COS9 cells: how do the findings from these studies translate to cardiac or skeletal muscle cells? The authors need to discuss this. Ideally, these studies should have been done in muscle cells, however this reviewer recognizes the technical difficulties of such experiments, although viral constructs can be effective. Moreover, coincident localization does not necessarily mean association. The authors should perform co-immunoprecipitation experiments of the combinations of overexpressed proteins described in Fig. 3B-D to validate the co-localization findings.

3. The SMDS observations will be more convincing if they are confirmed by atomic force microscopy studies of the TK construct(s).

Dear Dr D. Senyilmaz Tiebe,

We have submitted a revised version of our manuscript EMBOR-2019-48018V2. We thank you and the reviewers for the constructive comments to our original submission. We have carried out both additional experimentation as well as an in-depth revision of the text following these recommendations (modified text is shown in red in the revised manuscript to ease identification). In brief, we have performed pull-downs experiments from cell extracts, repeated the transfection work on a muscle-relevant cell line, deposited mass spectrometry data with a public repository and modified the written manuscript to better clarify the nature and novelty of our findings as well as to fit the journal format. I include below a detailed account of the changes implemented and answers to specific questions by the referees:

[1] *Referee #1: "The authors argue convincingly that titin is unlikely to be transported to protein degradation sites and that protein degradation is more likely to occur through the recruitment of ubiquitin binding protein. However, the motivation of the subsequent experiments is not clear as it only partially addresses the underlying scientific question."*

Our work focuses on the mechanism by which the sarcomere recruits its binding partners in a stretch dependent manner, thereby linking its content of functional and signaling factors to the mechanical demand on it exerted. While it is well established that the scaffolding properties of the sarcomere vary in function of mechanical activity, the molecular mechanisms by which such regulated scaffolding occurs remain largely unknown. A current hypothesis is that mechanical stretch induces conformational changes in sarcomere protein components that expose cryptic binding sites for functional factors. This mechanism was originally proposed to mediate the association of the autophagosomal receptors Nbr1 and p62 to M-line titin kinase in a stretch-dependent manner (*Lange et al, Science, 2005*). Unrelated to the topic of sarcomere-based interactions, independent reports have shown that protein components of the sarcomere - including titin- undergo ubiquitination in their sarcomeric context *in vivo* (*Witt et al, J Mol Biol, 2005; Wagner et al, Mol Cell Prot, 2012; Lang et al, Dis Model Mech, 2017*). However, a connection between ubiquitination and sarcomere-based interactions had not been made until now. In this work, we propose for the first time that <u>ubiquitination mediates sarcomere-based interactions and that the ubiquitination pattern of the sarcomere is sensitive to the mechanical state of the myofibril, in turn altering dynamically the protein associations that it supports.</u> In this regard, we show that the recruitment of Nbr1 and p62 to titin kinase occurs in non-stretched forms of the kinase once ubiquitination has taken place, so that the binding is not necessarily dependent on stretch-exposed cryptic binding sites but on post-translational modification. We also propose a mechanism by which ubiquitination could be modulated by stretch in the titin kinase locus. Our work is not directed to reveal the functional consequences of the recruitment of Nbr1/p62 to M-line titin (which we anticipate is a step within the muscle atrophy induced by MuRF1), but to explore the molecular mechanisms by which such regulated recruitment is exerted. We have modified Abstract, Introduction and Conclusion to better clarify to the reader the focus of our work and the novelty of the findings.

[2] *Referee #1: "Based on the EMBO manuscript submission guidelines, proteomics data should be submitted to a publicly accessible database, typically PRIDE. In addition, all identified sites should be documented in a suppl. table with relevant MS data to judge the quality of the MS data and modified peptides. The authors claim an absence of ubiquitylation in the D24728V mutant at a specific site but do not provide experimental evidence for this."*
Referee #1: "The authors describe that the A170-TKD24728V sample was subjected to ubiquitylation assays and modified lysine residues were identified by MS. The author should

show the results from three independent replicates with fold changes, statistical relevant values and MS data."

We thank the reviewer for pointing our attention to the PRIDE database. For each sample type (i.e. wild-type and D24728V), three independent biological replicates were generated and two samples from each were analysed (amounting to a redundancy of 6 measurements per sample type). The mass spectrometry data have been deposited with PRIDE under accession codes PXD015339 and PXD015340 (wild-type and D24728V samples, respectively). PRIDE provides access to those entries for review purposes, as follows:

entry PXD015339	Username: reviewer_pxd015339@ebi.ac.uk<br>Password: VtOGgfr9
entry PXD015340	Username: reviewer_pxd015340@ebi.ac.uk<br>Password: f3MfnVYp

[3] Referee #1: "A central claim of this manuscript describes titin not only as a sequestering protein, but also as a target of further modification with other functions. While the modification of titin is rather obvious, it is unclear what these new functions may be and the manuscript in its current form do not shed light onto this question. Ubiquitylation of titin may just change sequestration targets by creating binding sites for different types of proteins, which is per se not new."

To our knowledge, ubiquitination has not been proposed previously as a mechanism of sarcomere-based scaffolding and sarcomere ubiquitination has also not been proposed to be regulated by stretch. While sarcomere ubiquitination is dependent on the population of E3 ubiquitin ligases present in the cell at a given time, to our knowledge no previous proposal has been made that correlates the mechanical activity of the sarcomere with its targetability by such E3 Ub ligases. Our model proposes that the ubiquitination state of the sarcomere is the combined result of cell proteome (physiological cues) and mechanical cues derived from exercise.
Unraveling the functional outcomes of sarcomere ubiquitination and its promoted interactions is highly challenging and beyond the scope of this work. A possibility is that sarcomere compartmentation could cause the sequestration of Nbr1/p62 away from their place of action in the cell, thereby reducing cytoplasmic pools and down-regulating their activity. Alternatively, the targeting of the sarcomere M-line by Nbr1/p62 (possibly combined with the action of proteases) might contribute to sarcomere degradation. In line with that potential functional outcome, it is known that titin M-line is required for sarcomere integrity (*Weinert et al, J Cell Biol, 2006; Peng et al, Circulation, 2007; Radke et al, Circulation, 2019*), that MuRF1 promotes muscle atrophy and that Nbr1/p62 mediate the autophagy of large protein cargos. We have modified the Conclusion section to better discuss this point.

[4] *Referee #1: "Detailed characterization of the ubiquitylation site is advisable in particular with respect to protein stability. However, this might be a challenge for the giant protein titin and will be hard to address."*

We agree with the reviewer that measuring titin stability in the sarcomere as a function of the ubiquitination sites identified in the M-line is a staggering challenge. The huge size of the titin molecule (>3 MDa) and the fact that it is an intrasarcomeric protein makes the generation of mutagenized transgenic muscle samples and their subsequent analysis extraordinarily demanding. In addition, ubiquitination sites have been found in titin regions other than the M-line kinase. Thus, measuring the contribution of M-line ubiquitination to titin stability in a cellular or myofibrillar context is extremely difficult.

[5] *Referee #1: "The authors suggest that ubiquitylation recruits Nbr1 and p62 to titin and not stretch-induced exposure of binding sites. A supportive experiment would be useful to show that stretched titin (or equivalent construct) does not bind to Nbr1 or p62. Supporting evidence is lacking and co-localization studies with the unstretched A168-TK construct cannot exclude that Nbr1 and p62 are recruited to titin."*

Currently, no *in vitro* methodologies exist that permit applying stretch to a molecule along a defined molecular axis in conditions that are compatible with down-stream biochemical functional assays. Thus, the directionally stretched state of A168-TK could not be recreated in this study. The testing of a truncated form of titin kinase lacking its terminal tails, thought to mimic the stretched state, was reported by *Lange et al, Science, 2005.* That study and the current study are not in contradiction and the results are not mutually exclusive. Our proposed MuRF1-mediated mechanism is distinct from the stretched-promoted association previously proposed by Lange et al. In that previous work, forms of the kinase lacking the terminal tails interacted with Nbr1 through its PB1 domain. Lange et al did not observe association of Nbr1/p62 to full-length forms of TK containing the flanking tails. In full agreement with that result, our study of full-length TK also did not found an association with Nbr1/p62. However, we observe kinase association with both Nbr1 and p62 upon ubiquitination by MuRF1. This association is mediated by the UBA domains of Nbr1 and p62 (and not the PB1 domain). MuRF1 levels in muscle are strongly up-regulated by atrophic stimuli, while its levels are not significant in active muscle. Conceivably, these two distinct modes of Nbr1/p62 association with titin – in active and atrophic muscle, respectively – are not mutually exclusive but lead to different functional outcomes. It is tantalizing to envision that distint association modes might regulate the functionality of Nbr1/p62, as to differentiate *sequestration* from *targeting for degradation* outcomes. We have modified the Conclusion section to clarify this question to the reader.

[6] *Referee #1: "The authors propose that Nbr1 and p62 are recruited titin via their ubiquitin binding domain. Importantly, while the A168-TK showed diffuse expression, co-expression with Murf1 leads to A168-TK accumulation in large aggregates (Fig 3c) with remarkable shapes, suggesting for a change in localization of A168-TK itself. One obvious explanation is that A168-TK gets ubiquitylated and subsequently recruited to sites of protein degradation. Conversely, ubiquitin binders Nbr1 and p62 are recruited to these sites as well, independently of A168-TK. Co-localization is not sufficient to show binding of Nbr1 and p62 to A168-TK."*

We thank the reviewer for calling our attention to this possibility. In our opinion, existent data do not suggest a coincidental, independent localization of samples in the cell. The interaction of Nbr1/p62 with M-line titin was first observed in native myofibrils, where Nbr1/p62 were recruited to the non-diffusible sarcomeric M-line, giving a localization pattern that is otherwise atypical for these proteins (*Lange et al, Science, 2005*). Our cell experiments use a diffusible titin fragment, A168-TK, which remains diffuse in the cytoplasm when expressed in isolation. Overexpressed MuRF1 forms characteristic filamentous-like formations in the cell. Upon co-expression of A168-TK and MuRF1, A168-TK is recruited to MuRF1 formations producing a filamentous pattern which exactly agrees with that of MuRF1 (in agreement with the well-established and in vitro validated binding of these proteins; *Centner et al, JMB, 2001; Mrosek et al, FASEB J, 2007*). It can be predicted that the recruited A168-TK is ubiquitinated by the active MuRF1 in those formations (as observed in *in vitro* assays) and, furthermore, that even MuRF1 expressed in isolation is heavily auto-ubiquitinated (*in vitro* assays show that MuRF1 undergoes strong self-ubiquitination as it is characteristic of E3 ubiquitin ligases). Yet, neither the pattern of MuRF1 nor that of the MuRF1/A168-TK co-expressions resembles that observed

upon co-expression with Nbr1 or p62, which adopts the form of puncta. If MuRF1/A168-TK were recruited to puncta formations independently from Nbr1 and p62, the phenotypes would coincide, which is not the case. To address this point, we have expanded Figure 3 to display cell localization phenotypes for all proteins expressed in isolation as well as the A168-TK/MuRF1 complex in the absence of Nbr1/p62.

[7] *Referee #1: "It might be helpful to perform an immunoprecipitation experiment with ubiquitylation titin to identify the ubiquitylation-interaction of titin with Nbr1 and p62 via mass spectrometry."*

Referee #2: "Coincident localization does not necessarily mean association. The authors should perform co-immunoprecipitation experiments of the combinations of overexpressed proteins described in Fig. 3B-D to validate the co-localization findings."

Stimulated by the referee comments and to test whether the interaction with Nbr1/p62 is mediated by the ubiquitin moieties introduced in A168-TK by MuRF1, we have performed additional experimentation. As suggested, we first attempted co-immunoprecipitation experiments from cell lysates. However, in repeated attempts, Nbr1 and p62 were consistently found in the insoluble pellet remaining after cell lysis, which also contained large amounts of MuRF1 and A168-TK. This result reflected the insolubility of the protein species under study: Nbr1 and p62 form filaments through their PB1 domain and their insolubility is documented (e.g. *Paine et al, FEBS Lett, 2005; Kirkin et al, Mol Cell, 2009; Zaffagnini et al, EMBO J, 2018*), similarly the MuRF1/A168-TK filament-like formations proved to have poor solubility. The insolubility of the samples could not be overcome in the presence of detergents that preserve non-covalent interactions and that are compatible with IP. Due to the persistent technical difficulties, we performed instead pull-down experiments using recombinant titin samples that had been MuRF1-ubiquitinated *in vitro* and repurified. Ubiquitinated titin samples were then used as bait to capture endogenous p62 and Nbr1 proteins from cell extracts. In this way, we could confirm that endogenous p62 binds selectively to the ubiquitinated titin kinase and not to the unmodified sample (new data provided as Fig EV3). Sadly, despite multiple attempts, good quality data could not be obtained for Nbr1. Those experiments were troubled by continued solubility problems and a persistent unspecific association of Nbr1 to affinity beads. Because of the technical difficulties posed by these samples, in this work we had constructed instead truncated Nbr1 and p62 that lacked ubiquitin binding domains (Nbr1-ΔUBA; p62-ΔUBA) and tested this in cell co-transfection experiments (Fig 3). Those results showed that UBA domain deficiency abrogated the colocalization of Nbr1 and p62 with titin A168-TK, indicating that the association is mediated by ubiquitin-binding. As expected, titin and MuRF1 continued to associate mutually undisturbed in the presence of the UBA-truncated Nbr1/p62 samples (Fig 3). Taken together, pull-down results on p62 and cell transfections with -ΔUBA Nbr1/p62 variants support the view that the interaction of the TK-MuRF1 complex with autophagy adaptors p62 and Nbr1 is ubiquitin-mediated. This is in good agreement with existing knowledge of the interaction of p62/Nbr1 with poly-ubiquitinated proteins (e.g. *Zaffagnini et al, EMBO J, 2018).*

[8] *Referee #1: "The authors point out correctly, that endogenous titin is static and rather immobile. However, experiments are done with small constructs covering only one part of titin, which is acceptable given the size of full length titin. However, the authors should address experimentally or carefully discuss, why ubiquitylation of a diffusible protein construct has the same or a similar effect compared to the ubiquitylation of the entire titin."*

The association of Nbr1 and p62 with titin kinase was originally discovered based on the localization of these proteins to the sarcomeric M-line in native myofibrils (*Lange et al, Science,*

2005). One other unrelated study used immunogold-EM to show the presence of ubiquitin epitopes in the sarcomere M-line and Western-blots to reveal that titin extracted from muscle fibers is ubiquitinated (*Witt et al, J Mol Biol, 2005*). The latter has been recently confirmed by proteomics studies on titin extracted from muscle samples (*Wagner et al, Mol Cell Proteomics, 2012; Lang et al, Dis Model Mech, 2017*). Thus, both the ubiquitination of titin *in situ* and the recruitment of Nbr1 and p62 to the sarcomere M-line have been observed in the natural myofibril context. However, a connection between these two events had not been made previously. Our study uses a multi-domain titin fragment to analyze the mechanistic basis of the association of Nbr1/p62 with the titin kinase region. While the titin fragment is soluble and can migrate in the cell, the mechanistic basis of the interaction at the molecular level can be expected to be retained as no alteration has been made to the protein. Results from this study show that the fragment continues to support both ubiquitination and protein interactions.

[9] *Referee #1: "The authors propose a dynamic regulation of ubiquitylation upon stretch, supported by SMD simulation analysis. However, in-vitro ubiquitination data (Fig2) show that stretch is not required for efficient ubiquitylation. This point should be at least discussed."*

As the reviewer correctly states, our work shows that the ubiquitination of titin kinase by MuRF1 occurs in the absence of stretch. Our postulate is that stretch would *decrease* the levels of ubiquitination in this titin region by distancing ubiquitination target sites from the docked MuRF1 protein. The distancing of the sites is based on SMD simulations (and in agreement with previous experimental AFM data; point [15]) that reveal the unraveling of the NL sequence (i.e. the linker between the MuRF1 docking site and the kinase fraction) as the first unfolding event. We have clarified better this point to the reader in the Results Section, pg10.

[10] *Referee #1: "For reader convenience, all abbreviations should be clarified. Fig.1A requires improvements. In principle, pieces of information that come up later in the manuscript should be displayed in this panel. This includes: (i) all motifs (ii) the localization of detected ubiquitylation sites (iii) an overview of full length titin, which certainly helps to better orient within this huge protein."*

Following referee recommendation, we give the full definition of each abbreviation at the time of first occurrence in the text as well as facilitate the reading by minimizing the use of abbreviations, there where possible.
We have also expanded Fig 1A to include an overview of full-length titin, as requested. However, the original panel 1A aimed to provide the reader with a schematic overview of the domain composition of the kinase region of titin, as to make the reader familiar with its domain nomenclature. The inclusion of those abundant data in this graph makes the Figure panel excessively crowded and complex. We feel that such extensively annotated panel no longer serves the reader well. On the other hand, motifs and ubiquitination sites are appropriately illustrated in subsequent figures (namely, Fig 1C and Fig 2). Thus, we kindly request that Fig 1A is considered as provided in this updated version.

[11] *Referee #1: "The authors are advised to consistently use the term "mass spectrometry" rather than "mass spectroscopy". In addition, the authors may discuss how many ubiquitylation sites are unknown and which of them have been observed in in-vivo studies using the given reference. An overlap to known sites from the phosphosites.org database could also be useful."*

We thank the referee for calling our attention to the inconsistent usage of the terms spectrometry/spectroscopy. We now use “mass spectrometry” throughout.
In an attempt to focus our work on the ubiquitination mediated by MuRF1, we originally narrowed the comparison of our *in vitro* identified sites to those found *in vivo* by Lang et al (*Dis Model Mech, 2017)*. Lang et al used denervated mouse hind-limb as atrophic muscle model, where predictably MuRF1 levels were significant. We are only aware of one other study aimed to identify MuRF1 ubiquitination sites in myofibrils with residue precision (*Rubel et al, Cell Biochem Biophys, 2013*). However, the methodology used in that work relied on substrates being soluble under mild conditions, a requisite that sarcomeric filaments do not meet. Nevertheless, encouraged by the reviewer’s comment, we have expanded our comparison to the ubiquitination sites in titin reported by Wagner et al (*Mol Cell Proteomics, 2012*), who studied non-atrophic muscle and where identified Ub sites cannot be attributed to specific E3 ligases. The ubiquitination sites in the titin kinase region reported in the phosphosite.org database are those in the study by Wagner et al, 2012 (the results by Lang et al are not present in the database). It must be noticed that the two *in vivo* studies agree in only 3 Ub-sites out of the 9 sites observed in total. Such differences are indeed expected as the studies used different muscle types (Wagner et al used unspecified skeletal and heart muscle; Lang et al used gastrocnemius muscle) and in different physiological states (Wagner et al used healthy muscle, while Lang et al used denervated, atrophic muscle), which would have resulted in different populations of E3 Ub ligases being present in the cell. The comparison of our *in vitro* identified sites to those reported *in vivo* revealed that out of 12 *in vitro* sites, 5 sites coincided with those *in vivo* – this is a good result, particularly when considering the low mutual agreement between *in vivo* studies. Overall, more sites might have been observed *in vitro* due to an increased occupancy of individual sites under laboratory reaction conditions, which facilitate detection. Alternatively, some sites might not be accessible in the *in vivo* sarcomere context. In any case, the low agreement of *in vivo* results emphasizes the complexity of such comparisons, particularly when the identity and abundance of specific E3 Ub ligases in the *in vivo* samples analyzed is unknown. As requested, we now present an expanded analysis of *in vivo* Ub sites (pg 6) and provide a graphical comparison in Fig 2.

[12] *Referee #1: “The authors shifted several important findings to the supplement, which makes the manuscript difficult to follow. Along these lines, some experiments were obviously performed, but not described or discussed in the manuscript.”*

We thank the reviewer for making us aware of this problem. To facilitate the information flow and better fit the journal’s format, we have now promoted 5 Appendix Figures to the Expanded View (Fig EV1-EV5). We hope that this eases the access to that information by the readers.

[13] Referee #2: “Ubiquitination experiments: Given that these experiments were done in vitro, the authors need to comment on how confident they are about all the identified sites possibly undergoing ubiquitination in vivo. Also, were the common sites identified in both constructs by mass spec? This needs to be clarified/included.”

For the possible occurrence of the observed sites *in vivo*, see response to point [11]. Regarding Ub site identification, this used mass spectrometry in all cases (see response to point [2]).

[14] Referee #2: “Overexpression studies in COS9 cells: how do the findings from these studies translate to cardiac or skeletal muscle cells? The authors need to discuss this. Ideally, these studies should have been done in muscle cells, however this reviewer recognizes the technical difficulties of such experiments, although viral constructs can be effective.”

We are most thankful to the reviewer for acknowledging the substantial technical difficulty of carrying such transfection studies in muscle cell lines. Nevertheless, we do recognize the importance of the question and, thus, have repeated the *in cellula* work using the H9C2 myoblast cell line. This cell line was originally isolated from BDIX rat ventricular tissue and is widely established as an *in vitro* model for skeletal and cardiac muscle. Although H9C2 cells do not have structured sarcomeres, they retain the fundamental biochemical, morphological, electrophysiological and molecular patterns of striated muscle. These include, but are not limited to, a muscle-like creatine phosphokinase isoenzyme profile, sensitivity to acetylcholine, cardiac L-type calcium current sensitive to isoproterenol, an overall muscle-like transcriptome and expression of cardiac muscle specific proteins (*Kimes & Brandt, 1976, Exp Cell Res; Hescheler et al, 1991, Circ Res; Branco et al, 2015, PLoS One*). Our new results in H9C2 cells reproduce and even enhance the results obtained previously in COS9 cells. We provide these new data as a revised version of Figure 3.

[15] Referee #2: "The SMDS observations will be more convincing if they are confirmed by atomic force microscopy studies of the TK construct(s)."

Atomic Force Microscopy data of the A168-M2 multi-domain segment of titin, which comprises the A170-TK segment used in SMD simulations in our study, have been previously reported (*Puchner et al, PNAS, 2008; Stahl et al, Biophys J. 2011*). Consistently, the AFM data revealed a first low-force unfolding event (hierarchically corresponding to the unfolding of the mechanically weakest element) that corresponds to the unravelling of a sequence of ~9.1 nm length. At the time of those studies, only an incomplete crystal structure of titin kinase (TK) was available, containing the C-terminal tail extension (CRD), but lacking the N-terminal segment (NL). Thus, that initial unfolding event could not be attributed to specific titin molecular features and it remained unexplained, although Puchner et al 2008 speculated that it could correspond to the unfolding of a sequence N-terminal to TK. The authors then placed the focus on studying the higher-force response of the CRD tail, which could be studied based on structural data available at that time. The SMDS calculations in our study use the complete structure of TK (reported in this manuscript) containing N- and C-terminal flanking tails, and permit now attributing the first force peak in experimental AFM traces to the dissolution of the NYD 'knot' motif. Thus, our simulations are in excellent agreement with AFM observations and explain now their molecular basis. We provide now an explanation of this point in pg 9.

In addition to addressing the specific referee points, we have also performed an overall revision of the manuscript format to adapt it to journal guidelines.

Finally, we would like to express our gratitude to the referees for their comments, which have undoubtedly contributed to the improvement of this manuscript. We hope to have addressed the issues raised satisfactorily in this letter and in the revised version of the text and, thus, that EMBO Reports is willing to consider our work further. In this hope, we remain expectant.

Yours sincerely,

Olga Mayans
(On behalf of all authors)

1st Revision - Editorial Decision 27th May 2021

Dear Olga,

Thank you for submitting your revised manuscript. It has now been seen by both of the original referees.

As you can see, the referees find that the study is significantly improved during revision and recommend publication. However, I need you to address the editorial points below before I can accept the manuscript:

• Please make the PXD015339 and PXD015340 datasets public.
• As per our format requirements, in the reference list, citations should be listed in alphabetical order and then chronologically, with the authors' surnames and initials inverted; where there are more than 10 authors on a paper, 10 will be listed, followed by 'et al.'. Please see https://www.embopress.org/page/journal/14693178/authorguide#referencesformat
• Please upload the figures in one of the following formats: TIFF, PDF or EPS (please see https://www.embopress.org/page/journal/14693178/authorguide#figureformat for more information).
• Please upload the EV figures as individual files.
• As per our format requirements, the title length cannot exceed 100 characters (including spaces). The title is currently too long.
• We note the following about the figure callouts: Fig 4C+D callouts, Fig EV5 panel callouts and Appendix Figure S3 callouts are missing.
• The movies need to be renamed as Movie EV#. Please update the callouts in the text accordingly.
• The movies need to be ZIPped with their legends.
• The movie legends should be removed from the Appendix file
• Table 1 should come at the end of the Manuscript file.
• The figure legends should follow-on from the References.

Thank you again for giving us to consider your manuscript for EMBO Reports, I look forward to your minor revision.

Kind regards,

Deniz

--
Deniz Senyilmaz Tiebe, PhD
Editor
EMBO Reports

Referee #1:

The authors have addressed all of the reviewer's concerns.

Referee #2:

The authors have addressed the majority of the previous criticisms successfully, and explained technical difficulties that prohibited them from addressing the remaining points. Moreover, they

have extensively edited their manuscript to include key discussion and interpretation points brought up during the original submission of the study.

Dear Dr D. Senyilmaz Tiebe,

We have been absolutely delighted to learn that the journal has given consideration to our revised manuscript and that this has been well received by the reviewers.

We apologize for the shortcomings of the documents we provided and that these did not fulfil the journal's requirements. We supply now amended versions of text and figures following the editorial points, as requested:

• *Please make the PXD015339 and PXD015340 datasets public*
We confirm that these entries are now publicly available in the PRIDE database.

• *As per our format requirements, in the reference list, citations should be listed in alphabetical order and then chronologically, with the authors' surnames and initials inverted; where there are more than 10 authors on a paper, 10 will be listed, followed by 'et al.'. Please see https://www.embopress.org/page/journal/14693178/authorguide#referencesformat.*

The reference list has been revised as to comply with journal requirements.

• *Please upload the figures in one of the following formats: TIFF, PDF or EPS (please see https://www.embopress.org/page/journal/14693178/authorguide#figureformat for more information).*
Figures have been uploaded as EPS files.

• *Please upload the EV figures as individual files.*
EV Figures have been uploaded as individual files.

• *As per our format requirements, the title length cannot exceed 100 characters (including spaces). The title is currently too long.*

The title has been changed to "Titin kinase ubiquitination aligns autophagy receptors with mechanical signals in the sarcomere" (95 characters, incl. spaces)

• *We note the following about the figure callouts: Fig 4C+D callouts, Fig EV5 panel callouts and Appendix Figure S3 callouts are missing.*

Callouts to Fig 4C can now be found in pg 8, to Fig 4D in pg 12, to Fig EV5 in pg 12 and to Appendix Fig S3 in pg 11.

• *The movies need to be renamed as Movie EV#. Please update the callouts in the text accordingly.*
The corrections have been implemented.

• *The movies need to be ZIPped with their legends.*
The change has been made.

• *The movie legends should be removed from the Appendix file*
The change has been made.

• *Table 1 should come at the end of the Manuscript file.*
The change has been made.

• *The figure legends should follow-on from the References.*
The change has been made.

We have tried our best to follow closely the guidelines for authors available on-line. We hope to have remedied the problems in our text and remain hopeful of your decision on our manuscript.

Yours sincerely,

Olga Mayans

2nd Revision - Editorial Decision 19th Jul 2021

Dear Olga,

Thank you for submitting your revised manuscript. I have now looked at everything and all is fine. Therefore, I am very pleased to accept your manuscript for publication in EMBO Reports.

Congratulations on a nice work!

Kind regards,

Deniz
--
Deniz Senyilmaz Tiebe, PhD
Editor
EMBO Reports

--

Please note that under the DEAL agreement of German scientific institutions with our publisher Wiley, you could be eligible for free publication of your article in the open access format. Please contact either the administration at your institution or our publishers at Wiley (emboreports@wiley.com) for further questions.

--

At the end of this email I include important information about how to proceed. Please ensure that you take the time to read the information and complete and return the necessary forms to allow us to publish your manuscript as quickly as possible.

As part of the EMBO publication's Transparent Editorial Process, EMBO reports publishes online a Review Process File to accompany accepted manuscripts. As you are aware, this File will be published in conjunction with your paper and will include the referee reports, your point-by-point response and all pertinent correspondence relating to the manuscript.

If you do NOT want this File to be published, please inform the editorial office within 2 days, if you have not done so already, otherwise the File will be published by default [contact: emboreports@embo.org]. If you do opt out, the Review Process File link will point to the following statement: "No Review Process File is available with this article, as the authors have chosen not to make the review process public in this case."

Should you be planning a Press Release on your article, please get in contact with emboreports@wiley.com as early as possible, in order to coordinate publication and release dates.

Thank you again for your contribution to EMBO reports and congratulations on a successful publication. Please consider us again in the future for your most exciting work.

****

THINGS TO DO NOW:

You will receive proofs by e-mail approximately 2-3 weeks after all relevant files have been sent to our Production Office; you should return your corrections within 2 days of receiving the proofs.

Please inform us if there is likely to be any difficulty in reaching you at the above address at that time. Failure to meet our deadlines may result in a delay of publication, or publication without your corrections.

All further communications concerning your paper should quote reference number EMBOR-2019-48018V3 and be addressed to emboreports@wiley.com.

Should you be planning a Press Release on your article, please get in contact with emboreports@wiley.com as early as possible, in order to coordinate publication and release dates.

EMBO PRESS

YOU MUST COMPLETE ALL CELLS WITH A PINK BACKGROUND

PLEASE NOTE THAT THIS CHECKLIST WILL BE PUBLISHED ALONGSIDE YOUR PAPER

Corresponding Author Name: Olga Mayans	
Journal Submitted to: EMBO Reports	
Manuscript Number: EMBOR-2019-48018V1	

Reporting Checklist For Life Sciences Articles (Rev. June 2017)

This checklist is used to ensure good reporting standards and to improve the reproducibility of published results. These guidelines are consistent with the Principles and Guidelines for Reporting Preclinical Research issued by the NIH in 2014. Please follow the journal's authorship guidelines in preparing your manuscript.

A- Figures
1. Data
The data shown in figures should satisfy the following conditions:

- the data were obtained and processed according to the field's best practice and are presented to reflect the results of the experiments in an accurate and unbiased manner.
- figure panels include only data points, measurements or observations that can be compared to each other in a scientifically meaningful way.
- graphs include clearly labeled error bars for independent experiments and sample sizes. Unless justified, error bars should not be shown for technical replicates.
- if n< 5, the individual data points from each experiment should be plotted and any statistical test employed should be justified
- Source Data should be included to report the data underlying graphs. Please follow the guidelines set out in the author ship guidelines on Data Presentation.

2. Captions
Each figure caption should contain the following information, for each panel where they are relevant:

- a specification of the experimental system investigated (eg cell line, species name).
- the assay(s) and method(s) used to carry out the reported observations and measurements
- an explicit mention of the biological and chemical entity(ies) that are being measured.
- an explicit mention of the biological and chemical entity(ies) that are altered/varied/perturbed in a controlled manner.
- the exact sample size (n) for each experimental group/condition, given as a number, not a range;
- a description of the sample collection allowing the reader to understand whether the samples represent technical or biological replicates (including how many animals, litters, cultures, etc.).
- a statement of how many times the experiment shown was independently replicated in the laboratory.
- definitions of statistical methods and measures:
  - common tests, such as t-test (please specify whether paired vs. unpaired), simple χ2 tests, Wilcoxon and Mann-Whitney tests, can be unambiguously identified by name only, but more complex techniques should be described in the methods section;
  - are tests one-sided or two-sided?
  - are there adjustments for multiple comparisons?
  - exact statistical test results, e.g., P values = x but not P values < x;
  - definition of 'center values' as median or average;
  - definition of error bars as s.d. or s.e.m.

Any descriptions too long for the figure legend should be included in the methods section and/or with the source data.

In the pink boxes below, please ensure that the answers to the following questions are reported in the manuscript itself. Every question should be answered. If the question is not relevant to your research, please write NA (non applicable). We encourage you to include a specific subsection in the methods section for statistics, reagents, animal models and human subjects.

USEFUL LINKS FOR COMPLETING THIS FORM

http://www.antibodypedia.com
http://1degreebio.org
http://www.equator-network.org/reporting-guidelines/improving-bioscience-research-repor
http://grants.nih.gov/grants/olaw/olaw.htm
http://www.mrc.ac.uk/Ourresearch/Ethicsresearchguidance/Useofanimals/index.htm
http://ClinicalTrials.gov
http://www.consort-statement.org
http://www.consort-statement.org/checklists/view/32-consort/66-title
http://www.equator-network.org/reporting-guidelines/reporting-recommendations-for-tumo
http://datadryad.org
http://figshare.com
http://www.ncbi.nlm.nih.gov/gap
http://www.ebi.ac.uk/ega
http://biomodels.net/
http://biomodels.net/miriam/
http://jjj.biochem.sun.ac.za
http://oba.od.nih.gov/biosecurity/biosecurity_documents.html
http://www.selectagents.gov/

B- Statistics and general methods

Please fill out these boxes (Do not worry if you cannot see all your text once you press return)

1.a. How was the sample size chosen to ensure adequate power to detect a pre-specified effect size?	NA
1.b. For animal studies, include a statement about sample size estimate even if no statistical methods were used.	NA
2. Describe inclusion/exclusion criteria if samples or animals were excluded from the analysis. Were the criteria pre-established?	NA
3. Were any steps taken to minimize the effects of subjective bias when allocating animals/samples to treatment (e.g. randomization procedure)? If yes, please describe.	NA
For animal studies, include a statement about randomization even if no randomization was used.	NA
4.a. Were any steps taken to minimize the effects of subjective bias during group allocation or/and when assessing results (e.g. blinding of the investigator)? If yes please describe.	NA
4.b. For animal studies, include a statement about blinding even if no blinding was done	NA
5. For every figure, are statistical tests justified as appropriate?	NA
Do the data meet the assumptions of the tests (e.g., normal distribution)? Describe any methods used to assess it.	NA
Is there an estimate of variation within each group of data?	NA

Is the variance similar between the groups that are being statistically compared?	NA

C- Reagents

6. To show that antibodies were profiled for use in the system under study (assay and species), provide a citation, catalog number and/or clone number, supplementary information or reference to an antibody validation profile. e.g., Antibodypedia (see link list at top right), 1DegreeBio (see link list at top right).	SQSTM1/p62 Rabbit mAb #5114S (Cell Signalling Technology); NBR1 (D2E6) Rabbit mAb #9891 (Cell Signalling Technology); Anti-rabbit IgG, HRP-linked Antibody #7074 (Cell Signalling Technology)
7. Identify the source of cell lines and report if they were recently authenticated (e.g., by STR profiling) and tested for mycoplasma contamination.	H9c2(2-1) (ATCC® CRL-1446™) and COS-7 cells (ATCC® CRL-1651TM) cell lines were obtained from ATCC, autenthicated and certified to be mycoplasma free. Cells were grown in our laboratory for not more than 5 passages in medium supplemented with Normocin™ a formulation of three antibiotics active against mycoplasma, bacteria and fungi.

* for all hyperlinks, please see the table at the top right of the document

D- Animal Models

8. Report species, strain, gender, age of animals and genetic modification status where applicable. Please detail housing and husbandry conditions and the source of animals.	NA
9. For experiments involving live vertebrates, include a statement of compliance with ethical regulations and identify the committee(s) approving the experiments.	NA
10. We recommend consulting the ARRIVE guidelines (see link list at top right) (PLoS Biol. 8(6), e1000412, 2010) to ensure that other relevant aspects of animal studies are adequately reported. See author guidelines, under 'Reporting Guidelines'. See also: NIH (see link list at top right) and MRC (see link list at top right) recommendations. Please confirm compliance.	NA

E- Human Subjects

11. Identify the committee(s) approving the study protocol.	NA
12. Include a statement confirming that informed consent was obtained from all subjects and that the experiments conformed to the principles set out in the WMA Declaration of Helsinki and the Department of Health and Human Services Belmont Report.	NA
13. For publication of patient photos, include a statement confirming that consent to publish was obtained.	NA
14. Report any restrictions on the availability (and/or on the use) of human data or samples.	NA
15. Report the clinical trial registration number (at ClinicalTrials.gov or equivalent), where applicable.	NA
16. For phase II and III randomized controlled trials, please refer to the CONSORT flow diagram (see link list at top right) and submit the CONSORT checklist (see link list at top right) with your submission. See author guidelines, under 'Reporting Guidelines'. Please confirm you have submitted this list.	NA
17. For tumor marker prognostic studies, we recommend that you follow the REMARK reporting guidelines (see link list at top right). See author guidelines, under 'Reporting Guidelines'. Please confirm you have followed these guidelines.	NA

F- Data Accessibility

18: Provide a "Data Availability" section at the end of the Materials & Methods, listing the accession codes for data generated in this study and deposited in a public database (e.g. RNA-Seq data: Gene Expression Omnibus GSE39462, Proteomics data: PRIDE PXD000208 etc.) Please refer to our author guidelines for 'Data Deposition'.<br><br>Data deposition in a public repository is mandatory for:<br>a. Protein, DNA and RNA sequences<br>b. Macromolecular structures<br>c. Crystallographic data for small molecules<br>d. Functional genomics data<br>e. Proteomics and molecular interactions	Crystal structure coordinates and experimental structure factors have been deposited with the Protein Data Bank (entry 6YGN). Script files for force-biased molecular dynamics simulations (including coordinate files, custom index files, and .mpd files used) have been deposited with Figshare (DOI: 10.6084/m9.figshare.9777620); the files allow for the reproduction of the simulations when using Gromacs 5. Mass spectrometry data and original source files have been deposited with the ProteomeXchange Consortium via the PRIDE partner repository with the dataset identifiers PXD015339 and PXD015340 for titin wild-type and D24728V samples, respectively.
19. Deposition is strongly recommended for any datasets that are central and integral to the study; please consider the journal's data policy. If no structured public repository exists for a given data type, we encourage the provision of datasets in the manuscript as a Supplementary Document (see author guidelines under 'Expanded View' or in unstructured repositories such as Dryad (see link list at top right) or Figshare (see link list at top right).	See 18.
20. Access to human clinical and genomic datasets should be provided with as few restrictions as possible while respecting ethical obligations to the patients and relevant medical and legal issues. If practically possible and compatible with the individual consent agreement used in the study, such data should be deposited in one of the major public access-controlled repositories such as dbGAP (see link list at top right) or EGA (see link list at top right).	NA
21. Computational models that are central and integral to a study should be shared without restrictions and provided in a machine-readable form. The relevant accession numbers or links should be provided. When possible, standardized format (SBML, CellML) should be used instead of scripts (e.g. MATLAB). Authors are strongly encouraged to follow the MIRIAM guidelines (see link list at top right) and deposit their model in a public database such as Biomodels (see link list at top right) or JWS Online (see link list at top right). If computer source code is provided with the paper, it should be deposited in a public repository or included in supplementary information.	NA

G- Dual use research of concern

22. Could your study fall under dual use research restrictions? Please check biosecurity documents (see link list at top right) and list of select agents and toxins (APHIS/CDC) (see link list at top right). According to our biosecurity guidelines, provide a statement only if it could.	NA
